# Epidemiology and outcomes of early-onset AKI in COVID-19-related ARDS in comparison with non-COVID-19-related ARDS: insights from two prospective global cohort studies

**DOI:** 10.1186/s13054-022-04294-5

**Published:** 2023-01-05

**Authors:** Bairbre A. McNicholas, Emanuele Rezoagli, Andrew J. Simpkin, Sankalp Khanna, Jacky Y. Suen, Pauline Yeung, Daniel Brodie, Gianluigi Li Bassi, Tai Pham, Giacomo Bellani, John F. Fraser, John Laffey, on behalf of the CCCC Consortium

**Affiliations:** 1 Department of Anaesthesia and Intensive Care Medicine, School of Medicine, Clinical Sciences Institute, University of Galway, Galway University Hospital, Saolta Hospital Group, Galway, H91 YR71 Ireland; 2 School of Medicine, University of Galway, Galway, Ireland; 3 School of Medicine and Surgery, University of Milano-Bicocca, Monza, Italy; 4 Department of Emergency and Intensive Care, San Gerardo University Hospital, Monza, Italy; 5 Queensland University of Technology, Brisbane, Australia; 6 CSIRO Australian e-Health Research Centre AU, Herston, Australia; 7 University of Queensland, Brisbane, Australia; 8 Department of Medicine, The University of Hong Kong and Queen Mary Hospital, Hong Kong, Hong Kong China; 9 Department of Medicine, Columbia College of Physicians and Surgeons, and Center for Acute Respiratory Failure, New-York-Presbyterian Hospital, New York, NY USA; 10 Service de Médecine Intensive-Réanimation, AP-HP, Hôpital de Bicêtre, Hôpitaux Universitaires Paris-Saclay, Le Kremlin-Bicêtre, France; 11 UVSQ, Inserm U1018, Equipe d’Epidémiologie Respiratoire Intégrative, Université Paris-Saclay, Villejuif, France

**Keywords:** Acute kidney injury, Cohort study, COVID-19, 28-day mortality, 90-day mortality, Outcome, ARDS

## Abstract

**Background:**

Acute kidney injury (AKI) is a frequent and severe complication of both COVID-19-related acute respiratory distress syndrome (ARDS) and non-COVID-19-related ARDS. The COVID-19 Critical Care Consortium (CCCC) has generated a global data set on the demographics, management and outcomes of critically ill COVID-19 patients. The LUNG-SAFE study was an international prospective cohort study of patients with severe respiratory failure, including ARDS, which pre-dated the pandemic.

**Methods:**

The incidence, demographic profile, management and outcomes of early AKI in patients undergoing invasive mechanical ventilation for COVID-19-related ARDS were described and compared with AKI in a non-COVID-19-related ARDS cohort.

**Results:**

Of 18,964 patients in the CCCC data set, 1699 patients with COVID-19-related ARDS required invasive ventilation and had relevant outcome data. Of these, 110 (6.5%) had stage 1, 94 (5.5%) had stage 2, 151 (8.9%) had stage 3 AKI, while 1214 (79.1%) had no AKI within 48 h of initiating invasive mechanical ventilation. Patients developing AKI were older and more likely to have hypertension or chronic cardiac disease. There were geo-economic differences in the incidence of AKI, with lower incidence of stage 3 AKI in European high-income countries and a higher incidence in patients from middle-income countries. Both 28-day and 90-day mortality risk was increased for patients with stage 2 (HR 2.00, *p* < 0.001) and stage 3 AKI (HR 1.95, *p* < 0.001). Compared to non-COVID-19 ARDS, the incidence of shock was reduced with lower cardiovascular SOFA score across all patient groups, while hospital mortality was worse in all groups [no AKI (30 vs 50%), Stage 1 (38 vs 58%), Stage 2 (56 vs 74%), and Stage 3 (52 vs 72%), *p* < 0.001]. The time profile of onset of AKI also differed, with 56% of all AKI occurring in the first 48 h in patients with COVID-19 ARDS compared to 89% in the non-COVID-19 ARDS population.

**Conclusion:**

AKI is a common and serious complication of COVID-19, with a high mortality rate, which differs by geo-economic location. Important differences exist in the profile of AKI in COVID-19 versus non-COVID-19 ARDS in terms of their haemodynamic profile, time of onset and clinical outcomes.

**Supplementary Information:**

The online version contains supplementary material available at 10.1186/s13054-022-04294-5.

## Background

Acute kidney injury (AKI) is a frequent and severe complication in the acute respiratory distress syndrome (ARDS) with AKI rates of 25–60% in mechanically ventilated patients with ARDS [1]. AKI in this population is associated with substantially reduced survival with shock being the predominant cause of death [1–3]. A wealth of studies published early on in the pandemic have described incidence and outcomes for AKI in patients with COVID-19, ranging from 28 to 46% depending on admission to ICU [4] with higher rates of kidney replacement therapy reported for European and American studies suggesting the impact of geo-economics status [4–7].

Similarities and differences in the causal relationship and pathophysiology for kidney injury in patients with ARDS and COVID-19 receiving invasive mechanical ventilation (IMV) likely exist. In ARDS, the use of injurious lung ventilation settings causes direct kidney tubular apoptosis in preclinical studies [8]. Differing disease patterns of kidney injury from acute tubular injury, rhabdomyolysis, and thrombotic microangiopathy to focal and segmental glomerulosclerosis exist in COVID-19 patients [9–13]. Given the tropism of severe acute respiratory syndrome coronavirus 2 (SARS-CoV-2) for angiotensin-converting enzyme 2, expressed within the kidney, direct cell injury remains a yet unproven possibility. Impaired coagulation and endothelialitis induced by SARS-CoV-2 might reduce renal perfusion and precipitate cell injury which is worsened with haemodynamic stress and inflammation induced by invasive mechanical ventilation [11].

Since January 2020, the COVID-19 Critical Care Consortium (CCCC) has generated a global data set on the demographics, management and outcomes of critically ill COVID-19 patients and includes patients that were managed before and after the introduction of disease-modifying therapies for COVID-19 including dexamethasone and tocilizumab in 2021 [14]. This consortium currently includes > 350 sites in over 48 countries [16]. We compared incidence and outcomes of AKI for patients with COVID-19-related ARDS to a cohort of patients with non-COVID-19 ARDS using the Large observational study to UNderstand the Global impact of Severe Acute respiratory FailurE (LUNG-SAFE) study. LUNG-SAFE was a pre-pandemic global cohort study undertaken in 459 ICUs from 50 countries across five continents [17]. We analysed the incidence and outcomes of KDIGO stages 1–3 AKI in COVID-19-induced ARDS in the early stages of IMV and compared this to a cohort with non-COVID-19 ARDS. Secondary objectives were to compare (a) the demographics; (b) illness severity patterns; (c) management approaches; and (d) the relationship between AKI, shock and outcome, in patients that develop AKI as part of COVID-19 or non-COVID-19 ARDS patients.

## Methods

### Study design and setting

We performed an analysis of the CCCC study database on patients who underwent IMV from February 2020 to June 2022, which at the time of this analysis had enrolled 18,964 patients with confirmed or suspected SARS-CoV-2 infection. The COVID-19 Consortium collaborates with the International Severe Acute Respiratory and Emerging Infection Consortium (ISARIC) group and their Short PeRiod IncideNce sTudy of Severe Acute Respiratory Infection (SPRINT-SARI) project [15]. The study protocol was approved by the Alfred Hospital Ethics Committee, Melbourne, Australia (Project: 62,066, Local reference: 108/20). Participating hospitals obtained local ethics committee approval, and a waiver of informed consent was granted in all cases. De-identified patient data were collected and stored via the Research Electronic Data Capture electronic data capture tool, hosted at the University of Oxford, Oxford, UK; University College Dublin, Dublin, Ireland; and Monash University, Melbourne, Victoria, Australia [16].

The LUNG-SAFE study was a global, multicentre prospective cohort study that enrolled 4499 patients with acute hypoxemic respiratory failure, including ARDS, undertaken in 459 ICUs from 50 countries in five continents prior to the pandemic, and has been described in detail elsewhere [17]. The LUNG-SAFE patient cohort with ARDS was used as a comparator group for this analysis.

### Participants

Patients enrolled in the COVID-19–CCCC database from February 2020 up to June 2022 with laboratory-confirmed (real-time polymerase chain reaction) or suspected diagnosis of SARS-CoV-2 infection, receiving IMV for any cause and with serum creatinine data available within 48 h of invasive mechanical ventilation, were enrolled. The LUNG-SAFE database consisted of adult patients enrolled in a 4-week inception period in the winter of 2014 to participating ICUs that were receiving IMV or non-invasive ventilation.

**Fig. 1:**
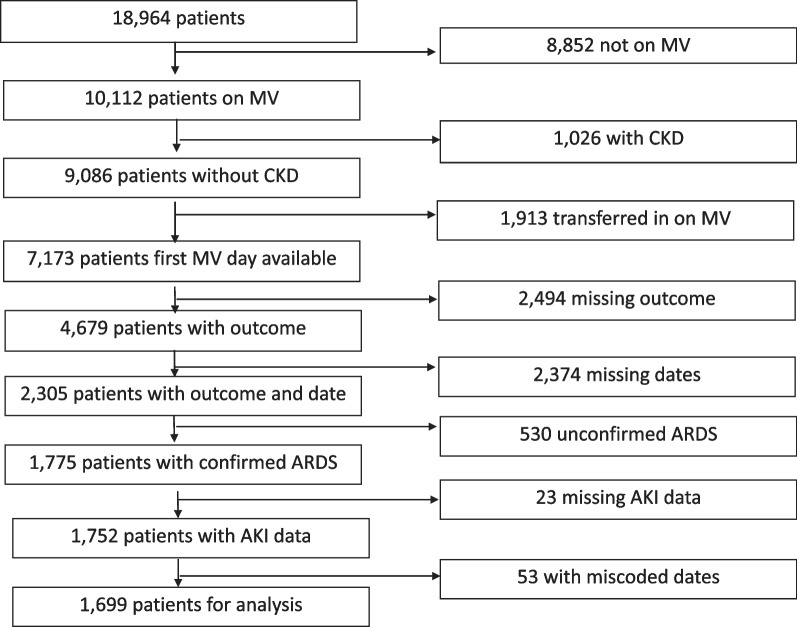
Flow chart for outcome analysis

For both the CCCC and LUNG-SAFE cohort, ARDS was defined using the ‘Berlin definition’ [18] and was confirmed to fulfil specific criteria for impaired oxygenation, the presence of new infiltrates on chest imaging, requirement for positive pressure ventilation, and known underlying cause. Patients with chronic kidney disease (CKD) prior to hospitalization (i.e. pre-existing glomerular filtration rate (GFR) of < 60 ml/min/1.73 m^2^) [19] patients transferred from other ICUs already undergoing IMV, patients with missing data for outcomes, dates (of admission, MV and death) and serum creatinine were excluded (Fig. 1, Additional file 1: Fig. S1). Chronic comorbidities in both cohorts included chronic respiratory impairment, congestive heart failure, chronic liver failure, immune incompetence and diabetes. Shock was defined as a cardiovascular SOFA score > 1 within the first 48 h of invasive mechanical ventilation.

### Study outcomes, data sources, measurements and definitions

As described in the CCCC study protocol [16] after enrolment, data on demographics, comorbidities, clinical symptoms and laboratory values were collected by clinical/research staff in all participating ICUs and recorded in an electronic case report form up to 28 days from commencement of IMV. Geo-economic regions were defined using the World Bank classification of gross national income into Europe, rest of the world high income and rest of the world middle income [20]. Primary outcomes in the CCCC study were all-cause mortality within 28 days of IMV and within 90 days of hospital admission.

### AKI classification criteria and data definition

For both cohorts, maximum serum creatinine (SCr) on day 1–2 of invasive mechanical ventilation was used to categorize patients in stages of AKI. Urine output was not used to define AKI, as it was not reliably documented in all patients. Kidney Disease Improving Global Outcomes (KDIGO) classification of AKI was used as follows: stage 1: ≥ 0.3 mg/dL or to > 1.5 to 2 × increase in Cr; stage 2: > 2 to ≤ 3 × increase in Cr; and stage 3: increase > 3 × increase in Cr or rise to ≥ 4.0 mg/dL or new initiation of renal replacement therapy (RRT) [21]. A baseline of GFR of 75 ml/min/1.73 m^2^ was assigned to back-calculate creatinine in patients in whom a baseline serum creatinine was not available as per KDIGO/AKI guidelines [21]. As ethnicity was not recorded in the LUNG-SAFE study, The Chronic Kidney Disease Epidemiology Collaboration (CKD-EPI) without race equation was used to estimate baseline serum creatinine using a presumption of a baseline serum creatinine > 0.7 mg/dl and > 0.9 mg/dl for females and males, respectively [22, 23].

### Statistical analysis

Both outcomes in our main analysis were time-to-event, whether patients died within 28 days of commencing invasive MV and whether patients died within 90 days of admission to hospital. Patients could die or be discharged before these dates or could be censored by still being on invasive MV at 28 days for example. To investigate the association of AKI with mortality, we used a Cox proportional hazards model with death within 28 days of commencing invasive MV as the primary outcome and repeated this using death within 90 days of hospitalization as the outcome [23]. In each model, we adjusted for age (years), sex, BMI (kg/m^2^), geo-economic region (Europe, rest of the world high income and rest of the world middle income), comorbidities (diabetes, hypertension, malignant tumours), respiratory failure severity (mild, moderate, severe), chronic cardiac disease, treatment with heparin, steroids or antibiotics, SOFA cardiovascular score, SOFA coagulation score, and the worst recorded PEEP, ECMO, tidal volume and respiratory rate measured in the first 48 h on invasive MV. These variables were chosen based on previous research on AKI and mortality in ICU [24–26]. To account for missing data in AKI and other covariates, we use multiple imputation with 10 data sets imputed, Cox models performed and hazard ratios (HR) from each model combined using Rubin’s rules [27]. We decided not to impute outcome data (outcome event or date). The list of missing variables is reported in Additional file 1: Fig. S7, and the analysis using only complete cases is reported Additional file 2: Tables S6 and S7. We report these combined HR along with 95% confidence intervals and *p* values. All analyses were carried out in R v4.1, and data are reported in accordance with STROBE Guidelines [28].

Comparisons between the CCCC and LUNG-SAFE cohorts were made by comparing summary statistics from each cohort. For categorical data, we used the chi-squared test for association, and for continuous data, we used independent samples t tests.

## Results

### COVID-19 ARDS population

From February 2020 to June 2022, the CCCC enrolled 18,964 patients with confirmed or suspected COVID-19 infection requiring IMV. Patients with a history of pre-existing CKD (*n* = 939), patients transferred to the study ICU on IMV (*n* = 1884), and patients with missing outcomes (*n* = 2500) or dates (admission; IMV commencement, ARDS confirmation, AKI; survival status, *n* = 2963) were excluded from the final analysis. The remaining 1699 patients with COVID-19-related ARDS undergoing IMV and with data on serum creatinine within 48 h constituted the study population (Fig. 1).

### Demographics, illness severity and risk factors for AKI

Of 1699 patients studied, 110 (6.5%) had stage 1, 94 (5.5%) had stage 2, 151 (8.9%) had stage 3 AKI, while 1344 (79.1%) had no AKI within 48 h of commencing IMV (Table 1).

**Table 1:** Characteristics, illness severity profile and outcomes of patients with COVID-19, acute respiratory distress syndrome (ARDS) stratified by the presence of acute kidney injury (AKI)

	*n*	No AKI^a^	Stage 1 AKI^a^	Stage 2 AKI^a^	Stage 3 AKI^a^	*p* value^b^
Acute kidney injury (AKI)	1699	1344 (79.1%)	110 (6.5%)	94 (5.5%)	151 (8.9%)	
Age (years)	1697	56 (15)	62 (15)	61 (14)	58 (15)	< 0.001
Sex (male)	1696	889 (66%)	71 (65%)	58 (62%)	94 (63%)	0.7
Body mass index (kg/m^2^)	1366	30 (8)	30 (8)	32 (10)	31 (9)	0.3
Region	1699					0.001
Europe high income		269 (20%)	15 (14%)	14 (15%)	15 (9.9%)	
Rest of the world high income		818 (61%)	83 (75%)	67 (71%)	100 (66%)	
Rest of the world middle income		257 (19%)	12 (11%)	13 (14%)	36 (24%)	
*Comorbidities*						
Diabetes	1652	388 (30%)	34 (33%)	32 (35%)	55 (38%)	0.2
Hypertension	1697	585 (44%)	72 (65%)	51 (54%)	91 (60%)	< 0.001
Malignant neoplasm	1696	52 (3.9%)	3 (2.8%)	4 (4.3%)	7 (4.6%)	0.9
Severe liver disease	1698	14 (1.0%)	2 (1.8%)	1 (1.1%)	1 (0.7%)	0.7
Chronic cardiac disease	1693	158 (12%)	29 (26%)	15 (16%)	27 (18%)	< 0.001
*Illness severity*						
ARDS severity category	1384					< 0.001
Mild		46 (4%)	2 (2%)	3 (4%)	4 (3%)	
Moderate		202 (19%)	21 (20%)	13 (16%)	23 (17%)	
Severe		818 (77%)	81 (78%)	65 (80%)	106 (80%)	
Sequential organ failure assessment (cardiovascular)	1552					
0		1033 (84%)	66 (65%)	59 (69%)	103 (76%)	< 0.001
1		185 (15%)	36 (35%)	26 (30%)	30 (22%)	
2		4 (0.3%)	0 (0%)	0 (0%)	0 (0%)	
3		4 (0.3%)	0 (0%)	0 (0%)	1 (0.7%)	
4		3 (0.2%)	0 (0%)	1 (1.2%)	1 (0.7%)	
Arterial pH	1453	7.34 (0.12)	7.26 (0.14)	7.22 (0.12)	7.23 (0.14)	< 0.001
*Management (worst parameters first 48 hrs)*						
Positive end-expiratory pressure (PEEP, cmH_2_O)	1316	11.6 (3.4)	12.0 (3.7)	12.1 (4.0)	11.4 (4.2)	0.6
Need for extracorporeal membrane oxygenation (ECMO)	1550	99 (8%)	13 (12%)	17 (18%)	18 (12%)	0.006
Lowest tidal volume (mL)	957	398 (121)	408 (125)	367 (131)	397 (140)	0.5
Highest respiratory rate (breaths per minute)	1290	26 (8)	27 (8)	27 (9)	28 (8)	< 0.001
*Treatments received*						
Heparin	886	636 (89%)	52 (88%)	40 (91%)	59 (86%)	0.8
Corticosteroids	1658	976 (75%)	69 (63%)	61 (65%)	98 (66%)	0.003
Antibiotics	1666	1267 (96%)	107 (99%)	89 (95%)	138 (93%)	0.084
*Outcomes*						
Length of stay on invasive mechanical ventilation (days) All	1654	12 (19)	12 (16)	10 (16)	7 (16)	< 0.001
Survivors	752	13 (20)	16 (24)	23 (30)	18 (17)	0.061
Length of stay in ICU (days) All	1528	22 (41)	10 (74)	20 (19)	17 (17)	0.003
Survivors	760	28 (45)	8 (101)	34 (19)	30 (21)	0.028
Length of stay in hospital All	1490	33 (40)	26 (33)	28 (26)	24 (24)	< 0.001
Survivors	735	43 (46)	39 (22)	54 (27)	46 (27)	0.018
28-day mortality	1699	544 (40%)	55 (50%)	62 (66%)	104 (69%)	< 0.001
90-day mortality	1699	660 (49%)	64 (58%)	70 (74%)	105 (70%)	< 0.001

^a^*n* (%); Mean (SD)

^b^Pearson's chi-squared test; Kruskal–Wallis rank-sum test; Fisher's exact test

Patients with AKI were older and more likely to have hypertension and chronic cardiac disease than patients without AKI (*p* < 0.001). There were geo-economic differences in the severity profile of AKI, with a lower proportion of stage 3 AKI in European high-income and a higher proportion in patients from the rest of the world middle-income countries (*p* < 0.001).

The proportion of patients with severe respiratory failure was higher in those patients with higher stages of AKI (Table 1). Respiratory rate was higher (*p* < 0.001), and pH was lower in patients with AKI (*p* < 0.001), but there was no difference in PEEP or tidal volume in patients with AKI compared to patients without AKI. The use for ECMO was highest in patients with stage 2 AKI and higher in all patients with AKI (*p* = 0.006). Receipt of corticosteroids was lower in patients with AKI and lowest in stage 3 AKI (*p* = 0.003) (Table 1).

Factors independently associated with the development of AKI were older age, hypertension, ECMO, higher respiratory rate and higher haemodynamic component of the SOFA score. Corticosteroids were associated with a reduced risk of development of AKI [adjusted OR = 0.799, 95% confidence interval (CI) = 0.671–0.950, *p* = 0.011] (Table 2). Geo-economic region (rest of world income vs Europe and middle income vs Europe) was associated with a risk for AKI development (Table 2). Incidence of AKI for each quarter throughout the pandemic is presented in Additional file 2: Table S8.

**Table 2:** Multivariable logistic regression model of factors associated with the development of acute kidney injury (AKI) (*n* = 1699 patients)

Variable	Odds ratio	95% confidence interval	*p* value
Corticosteroid therapy	0.74	0.63, 0.88	< 0.001
Age (years)	1.01	1.01, 1.02	< 0.001
Sex (male)	0.98	0.82, 1.16	0.78
Body mass index (kg/m^2^)	1.01	0.99,1.02	0.19
Region (world high income vs Europe)	1.40	1.14, 1.73	0.002
Region (world middle income vs Europe)	1.57	1.16, 2.12	0.003
Comorbidity diabetes	1.09	0.92,1.28	0.33
Hypertension	1.26	1.07, 1.48	0.01
Malignant neoplasm	1.05	0.73, 1.49	0.81
Cardiac disease	1.22	0.99, 1.50	0.06
Respiratory failure (moderate vs mild)	1.34	0.86, 2.10	0.20
Respiratory failure (severe vs mild)	1.33	0.87, 2.03	0.19
Positive end-expiratory pressure (PEEP, cm H_2_O)	1.00	0.98, 1.03	0.76
Extracorporeal membrane oxygenation (ECMO) support	1.75	1.37, 2.24	< 0.001
Tidal volume (mL)	1.00	0.99, 1.01	0.58
Respiratory rate (breaths per minute)	1.01	1.00. 1.03	0.01
Sequential organ failure assessment (platelets)	0.96	0.85,1.07	0.46
Sequential organ failure assessment (cardiovascular)	1.28	1.10,1.49	< 0.001

### Patient outcomes

**Fig. 2:**
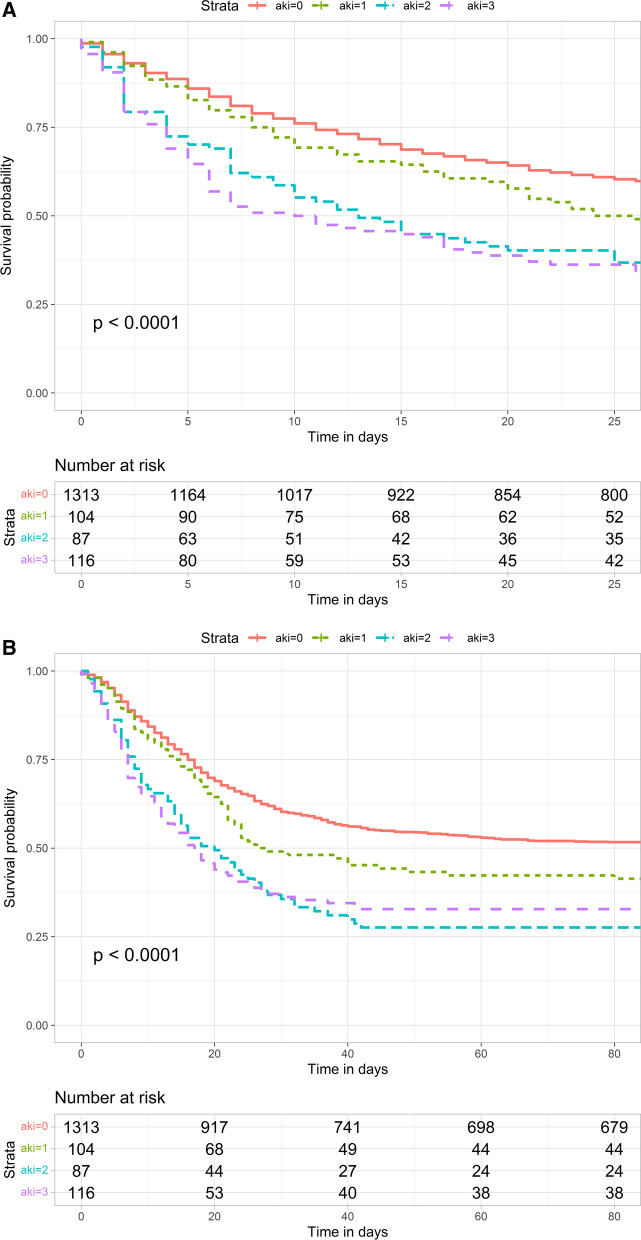
**A** Kaplan–Meier plot of 28-day hospital survival and AKI stage. **B** Kaplan–Meier plot of 90-day hospital survival and AKI stage

**Fig. 3:**
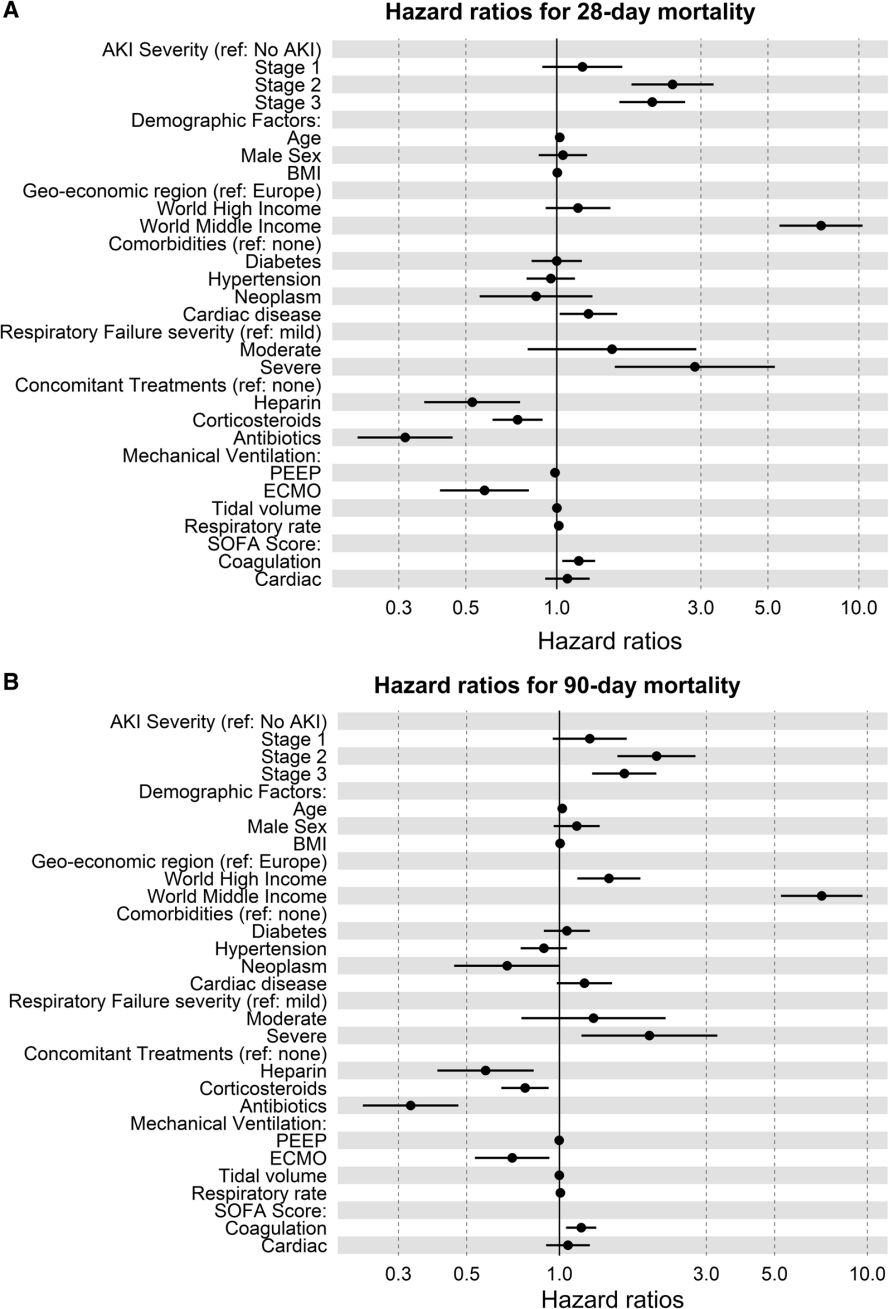
**A** Hazard ratio plots for 28-day ICU mortality against AKI stage. **B** Hazard ratio plots for 90-day hospital mortality against AKI stage

Outcomes in patients with AKI were worse compared to patients without AKI (Fig. 2A, B). For patients that survived, duration of mechanical ventilation was not significantly different with AKI, while days in ICU (*p* = 0.028) and hospital (*p* = 0.018) were higher (Table 1). Mortality rate was higher for each stage of AKI (Table 1). Both 28-day (Fig. 3A) and 90-day (Fig. 3B) hospital mortality risk was higher in patients with Stage 2 (adjusted HR = 2.0, *p* < 0.001 for day 28, adjusted HR = 1.7, *p* = 0.001 for day 90) and Stage 3 (adjusted HR = 1.9, *p* < 0.001 for day 28, adjusted HR = 1.5, *p* < 0.001 for day 90) of AKI (Figs. 2A, B and 3A, B; Additional file 2: Tables S4 and S5). In the adjusted model, age, patients from the rest of the world middle-income countries, chronic cardiac disease, severe respiratory failure, higher respiratory rate and higher coagulation component of SOFA were each associated with increased 28-day mortality. All of these factors except severe respiratory failure, chronic cardiac disease and respiratory rate were associated with 90-day mortality in the model. Geo-economic region of the rest of the world high income compared to Europe was associated with increased 90-day mortality [HR 1.5 (95% CI 1.1–1.8), *p* = 0.002]. Conversely, treatment with heparin, corticosteroids, antibiotics and ECMO was associated with a reduction in mortality at 28 and 90 days (Fig. 3A, B)*.* Inclusion of patients with chronic kidney disease in the cohort did not alter 28- or 90-day mortality (Additional file 1: Figs. S2–S4). Multivariable analysis for the risk of death including patients with a history of chronic kidney disease showed no major difference to the study cohort (Additional file 1: Figs. S5 and S6). Multivariable analysis for risk of death using geographic region rather than geo-economic region showed African and Asian countries to have high 28- and 90-day mortality with COVID-19 (Additional file 1: Figs. S8 and S9).

## Comparison of patients with COVID-19 to non-COVID-19 ARDS

The demographics, clinical characteristics, initial ventilatory management and outcomes of patients with COVID-19 were compared to a similar cohort of patients with non-COVID-19 ARDS using the LUNG-SAFE cohort (*n* = 1957) (Additional file 1: Fig. S1).

### AKI incidence and onset time course

Incidence of AKI within 48 h of IMV was lower in COVID-19 ARDS compared to non-COVID-19 ARDS, with an overall incidence of 21% in patients with COVID and 54% in patients without COVID-19, *p* < 0.001 for each stage (Table 3). The time course of onset of AKI also differed, with 56% (*n* = 355/629) of all AKI in COVID group and 89% (*n* = 873/976) of all AKI in LUNG-SAFE patients occurred in first 48 h (Additional file 2: Table S1). Overall, the rate of AKI was lower over a 28-day period for patients in COVID-19 ARDS compared to the non-COVID-19 ARDS [37.7% (*n* = 629) vs. 49.9% (*n* = 927) COVID-19 ARDS vs non-COVID-19 ARDS, *p* < 0.001] (Additional file 2: Table S1).

**Table 3:** Demographics and ventilatory settings of patients with COVID-19 versus non-COVID-19 ARDS stratified by acute kidney injury (AKI) stage

	COVID-19 ARDS CCCC cohort (*n* = 1699)^a^	Non-COVID-19 ARDS LUNG-SAFE cohort (*n* = 1947)^a^	*p* value^b^
AKI severity category			
No AKI	1344 (79.0)	1084 (55.4)	< 0.001
Stage 1 AKI	110 (6.5)	319 (16.3)	
Stage 2 AKI	94 (5.5)	194 (9.9)	
Stage 3 AKI	151 (9.0)	360 (18.4)	
Age (years)			
No AKI	56 (15)	57 (17)	0.129
Stage 1 AKI	62 (15)	64 (15)	0.229
Stage 2 AKI	61 (14)	66 (16)	0.007
Stage 3 AKI	58 (15)	61 (16)	0.044
Sex (male)			
No AKI	889 (66)	680 (63)	< 0.001
Stage 1 AKI	71 (65)	196 (61)	
Stage 2 AKI	58 (62)	119 (61)	
Stage 3 AKI	95 (63)	211 (59)	
Body mass index (kg/m^2^)			
No AKI	30 (8)	26 (7)	< 0.001
Stage 1 AKI	30 (8)	28 (14)	0.071
Stage 2 AKI	32 (10)	29 (8)	0.012
Stage 3 AKI	31 (9)	28 (7)	0.002
Chronic disease^d^			
No AKI	537 (40)	552 (51)	< 0.001
Stage 1 AKI	58 (53)	192 (60)	
Stage 2 AKI	45 (48)	110 (57)	
Stage 3 AKI	69 (46)	212 (59)	
*Ventilator settings*^c^			
Fraction of inspired oxygen (FiO_2_, %)			
No AKI	79 (22)	66 (23)	< 0.001
Stage 1 AKI	79 (20)	68 (23)	< 0.001
Stage 2 AKI	86 (21)	70 (24)	< 0.001
Stage 3 AKI	84 (20)	72 (23)	< 0.001
Tidal volume (mL)			
No AKI	398 (121)	508 (126)	< 0.001
Stage 1 AKI	408 (125)	497 (122)	< 0.001
Stage 2 AKI	367 (131)	523 (134)	< 0.001
Stage 3 AKI	397 (140)	496 (125)	< 0.001
Positive end-expiratory pressure (PEEP, cm H_2_O)			
No AKI	11.6 (3.4)	8.9 (3.4)	< 0.001
Stage 1 AKI	12.0 (3.7)	9.0 (3.2)	< 0.001
Stage 2 AKI	12.1 (4.0)	9.1 (3.3)	< 0.001
Stage 3 AKI	11.4 (4.2)	9.8 (3.8)	< 0.001
Respiratory rate (breaths per minute)			
No AKI	26 (8)	22 (11)	< 0.001
Stage 1 AKI	27 (8)	23 (6)	< 0.001
Stage 2 AKI	27 (9)	23 (7)	< 0.001
Stage 3 AKI	28 (8)	23 (6)	< 0.001
Peak pressure (cm H_2_O)			
No AKI	25.3 (6.0)	27.8 (8.2)	< 0.001
Stage 1 AKI	25.1 (5.8)	29.1 (8.5)	< 0.001
Stage 2 AKI	26.2 (9.2)	28.9 (8.3)	0.007
Stage 3 AKI	27.3 (6.1)	29.4 (9.3)	0.004
*P*/*F* ratio (%)			
No AKI	127 (84)	149 (63)	< 0.001
Stage 1 AKI	118 (62)	145 (63)	< 0.001
Stage 2 AKI	100 (51)	139 (65)	< 0.001
Stage 3 AKI	109 (77)	138 (62)	< 0.001
PaCO_2_ (mmHg)			
No AKI	51 (17)	49 (16)	0.003
Stage 1 AKI	47 (11)	50 (18)	0.041
Stage 2 AKI	57 (15)	49 (15)	< 0.001
Stage 3 AKI	57 (22)	49 (16)	< 0.001
pH			
No AKI	7.34 (0.12)	7.34 (0.10)	0.999
Stage 1 AKI	7.26 (0.15)	7.29 (0.12)	0.06
Stage 2 AKI	7.22 (0.12)	7.25 (0.13)	0.054
Stage 3 AKI	7.23 (0.14)	7.23 (0.14)	0.999
Sequential organ failure assessment (cardiovascular score)			
No AKI	0.18 (0.45)	2.14 (1.70)	< 0.001
Stage 1 AKI	0.35 (0.48)	2.65 (1.62)	< 0.001
Stage 2 AKI	0.35 (0.61)	3.11 (1.39)	< 0.001
Stage 3 AKI	0.27 (0.58)	3.29 (1.32)	< 0.001
Sequential organ failure assessment (platelet score score)			
No AKI	0.30 (0.75)	1.21 (1.46)	< 0.001
Stage 1 AKI	0.28 (0.64)	1.39 (1.44)	< 0.001
Stage 2 AKI	0.21 (0.54)	1.37 (1.40)	< 0.001
Stage 3 AKI	0.26 (0.60)	1.79 (1.43)	< 0.001
*Outcomes*			
Length of stay on invasive mechanical ventilation (days)			
No AKI	18 (23)	12 (14)	< 0.001
Stage 1 AKI	16 (14)	12 (13)	0.01
Stage 2 AKI	15 (17)	11 (12)	0.043
Stage 3 AKI	12 (13)	13 (15)	0.453
Length of stay in ICU (days)			
No AKI	22 (41)	15 (15)	< 0.001
Stage 1 AKI	10 (74)	14 (15)	0.575
Stage 2 AKI	20 (19)	12 (12)	< 0.001
Stage 3 AKI	17 (17)	16 (17)	0.544
ICU mortality			
No AKI	655 (49)	269 (25)	< 0.001
Stage 1 AKI	63 (58)	114 (36)	
Stage 2 AKI	68 (74)	104 (54)	
Stage 3 AKI	106 (72)	190 (53)	
Hospital mortality			
No AKI	674 (50)	318 (30)	< 0.001
Stage 1 AKI	64 (58)	126 (40)	
Stage 2 AKI	70 (74)	115 (59)	
Stage 3 AKI	106 (72)	209 (58)	

^a^*n* (%); Mean (SD)

^b^Pearson's chi-squared test (categorical); independent sample t test (continuous)

^c^Ventilator setting data are the worst scores in first 48 h of invasive mechanical ventilation

^d^Diabetes, pulmonary disease, immune incompetence, cardiac disease, severe liver disease

### Demographic profile, illness severity and ventilatory settings

Compared to non-COVID-19 ARDS, patients with COVID-19 ARDS with stage 2 and 3 AKI were older, with a higher proportion being male, a higher BMI and fewer baseline chronic conditions. For all patients, within the first 48 h of IMV, FiO_2_, PEEP and respiratory rates were higher, while peak pressure and tidal volumes were lower compared to patients with non-COVID-19 ARDS (Table 3).

PaO_2_–FiO_2_ ratio (PFR) was lower in patients with COVID-19 ARDS with lower values for each stage of AKI (118 ± 84 vs. 145 ± 63 mmHg for stage 1, 100 ± 51 vs. 139 ± 65 mmHg for stage 2, and 109 ± 77 vs. 138 ± 60 mmHg for stage 3) in COVID-19 ARDS compared to non-COVID-19 ARDS (*p* < 0.001). PaCO_2_ was higher for patients without AKI and stage 2 and 3 AKI, while it was lower in stage 1 AKI compared to non-COVID-19 ARDS. There was no difference in pH between the cohorts (Table 3). The worst SOFA cardiovascular (*p* < 0.001) and coagulation scores within 48 h of IMV (< 0.001) were markedly lower for COVID-19 patients with and without AKI compared to patients with non-COVID-19 ARDS. Shock was present much less frequently in patients with COVID-19 ARDS; in stage 3, AKI shock was present in 23.4% of patients with COVID-19 ARDS compared to 90.8% patients with non-COVID-19 ARDS within 48 h of IMV (Table 3, Additional file 2: Table S2).

### Patient outcomes

All patient groups with COVID-19, except patients with stage 3 AKI, had a longer duration of IMV compared to patients with non-COVID-19 ARDS. However, duration of ICU stay was not different for stage 1 AKI or stage 3 AKI but was longer for no AKI and stage 2 AKI in the COVID-19 ARDS cohort (Table 3). Overall, both ICU and hospital mortality was worse across all patient groups (*p* < 0.001) in COVID-19 ARDS compared to non-COVID-19 ARDS with a similar incremental increase in mortality with increasing stages of AKI found between the two cohorts (Table 3, Additional file 2: Table S3).

## Discussion

In this study across two global ARDS cohorts, we examined the outcomes from AKI in patients with COVID-19 within 48 h of commencing IMV. Our first major finding is that incidence of AKI within 48 h of IMV was substantially lower compared to a non-COVID-19 ARDS cohort. We focussed on the incidence of AKI within 48 h in IMV in patients without a history of CKD. Restricting our cohort to AKI within 48 h of IMV provided an opportunity to study the potential influence of mechanical ventilation on outcomes related to AKI, rather than other factors that influence the development of AKI later on in the course of illness, which may have different outcomes [29]. This also permitted a direct comparison with a non-COVID-19 ARDS cohort, namely the LUNG-SAFE cohort [24]. Timing of AKI differed between the cohorts, with a higher number of patients developing AKI later on in their ICU course in patients with COVID-19 ARDS compared to the non-COVID-19 ARDS. Outcomes differ based on timing of AKI in critical illness [30]. Inflammatory, haemodynamic and immune-related factors within both the lung and kidney may drive the development of AKI [31]. Longer periods of IMV may lead to increased risk of opportunistic infections, sepsis and exposure to nephrotoxic medications. Hospital-acquired intrinsic acute kidney injury was associated with a higher stage of AKI, greater need for RRT and worse outcome in a study on aetiology of AKI in hospitalized COVID-19 patients [13, 32, 33].

We next found that the use of corticosteroids was associated with a reduced risk of AKI and reduced mortality in patients with established AKI. Corticosteroids are established as a definitive treatment for COVID-19 ARDS [14]. The cohort spanned a period before and after routine use of corticosteroids in severe COVID-19 pneumonitis. Corticosteroids may have reduced inflammatory damage within the lung that leads to increased risk of kidney injury. Alternatively, steroids may act locally within the kidney to reduce inflammatory response to COVID-19 infection [13]. This suggests an important role for inflammation in driving AKI related to COVID-19 and the importance of disease-modifying treatments in addition to supportive care.

Our next finding was a much lower incidence of shock as measured by the cardiovascular SOFA score in COVID-19 ARDS. Although more severely hypoxic, patients with COVID-19 ARDS had lower cardiovascular and coagulation SOFA score at 48 h compared to non-COVID-19 ARDS. Our findings are consistent with other COVID-19 studies, where AKI was found to be pre-renal from dehydration and thirst which respond to judicious fluid management rather than high-dose vasopressors [32]. Despite a lower incidence, and consistent with non-COVID-19 ARDS, cardiovascular SOFA was an independent factor associated with early AKI [25].

Ventilatory management was different but consistent across each stage of AKI, with lower tidal volume, peak pressure and higher PEEP use in patients with COVID-19 ARDS compared to non-COVID-19 ARDS. There were no differences in tidal volume or PEEP between patients who developed AKI who had COVID-19. This is consistent with non-COVID-19 ARDS where only a higher FiO_2_ and a higher PEEP were associated with severe AKI [24].

Mortality rates were markedly higher for all patients with COVID-19 with a graded increase for each stage of AKI consistent with non-COVID-19 ARDS [34]. In the LUNG-SAFE cohort, mortality in patients with AKI was in part attributable to cardiovascular causes, reflected by increased haemodynamic subcomponent of the SOFA score in this population. Cause of death was not recorded for the cohort, but a large cohort study from Argentina found that refractory hypoxaemia is the most common cause of mortality in, followed by septic shock and multi-organ dysfunction syndrome, with patients often having more than one cause of death [35]. Other studies have found that mortality was not strongly linked with increased lactate or mean arterial pressure [35, 36].

Overall, the rate of AKI in the COVID-19 cohort is lower than that reported in other studies examining AKI in COVID-19 patients undergoing IMV [4]. Geo-economic differences exist in the incidence and mortality rates in COVID-19 patients with AKI which may be related to resource availability or underlying genetic susceptibility. Geographic factors may have influenced the risk for and management of AKI in patients. This has biological plausibility in susceptibility given the existence of AE2 genetic polymorphisms between Asian and non-Asian populations [37]. Economic differences may also have influenced outcomes from AKI, with resource-poor countries not being able to support patients on dialysis and withdrawal of life-sustaining therapies possibly higher with AKI developing. In studies from USA from ICU’s early on in the pandemic, over half patients had onset of AKI within 24 h of intubation with most requiring RRT within 72 h of intubation [5, 34]. The lower rates in our study may reflect the longer time period that encompasses introduction of disease-modifying treatments such as dexamethasone and Il-6 blockers that were associated with a reduction in both respiratory and renal injury. Additionally, focussing on AKI within 48 h of intubation and excluding patients with chronic kidney disease also resulted in a population with a lower risk of AKI. It is unlikely that mis-classification of AKI using the updated CKD-EPI without race equation is a cause for a lower rate of AKI given that incidence of AKI was higher (45% vs 39%) using this staging system compared to that used in the LUNG-SAFE cohort [24].

Strengths of the study include the fact that this is an international large group of mechanically ventilated COVID-19 patients with ARDS, while the comparator non-COVID-19 group is also a global cohort. The wide geographic spread of participating ICUs and the large patient sample size permit a global view of the impact of AKI on outcomes in patients with COVID-19 undergoing IMV, but countries from low income geo-economic regions were not represented. The strict application of Strobe guidelines is also a strength. However, the observational nature of the study precludes the drawing of any causal inferences. While the possibility of immortal time bias related to the development of AKI and increasing severity of AKI throughout time exists, this is considered less likely to be important given that we focussed on early AKI within 48 h of IMV rather than at later time points. Potential heterogeneity exists in the criteria for admission to ICU; indication for IMV and use of adjuncts such as prone ventilation and neuromuscular blockade were not standardized across countries and could have depended on local practices. Another strength of this study is that we made use of the recently reported CKD-EPI equation without race to back-calculate serum creatinine from an assigned GFR when baseline serum creatinine is not available as part of categorizing stage of AKI [22]. An advantage to this approach is that the same classification system could be used across both cohorts allowing a direct comparison of the incidence of AKI. A disadvantage is that experience with application of the new equation outside of US populations is limited with loss of accuracy in Asian populations [38].

There are several limitations to the study. First is the high number of patients excluded due to missing data on timing and outcomes, which may have introduced a bias in the patients studied in the cohort. The extent of missing data is reported, and variables including outcomes, region and comorbidities had no or low missingness. Data on mechanical ventilatory variables and BMI had the highest degree of imputation but are consistent with that reported for non-COVID-19 which lends to the plausibility of the findings. With the exception of AKI stage 1, which was not associated with mortality outcomes, the direction of effect for all other variables using complete cases is consistent with the imputed data. As participation in the study was voluntary, a centre selection bias might be present. The data used are taken from routine clinical records; hence, missing data could bias estimates. Potential temporal trends in the treatment of COVID-19 may have influenced the findings, although where possible this was accounted for (e.g. use of Dexamethasone and other specific therapies). Logistical limitations likely existed related to managing critically ill patients treated in newly developed and understaffed ICUs early in the pandemic, due to shortages in CCRT devices. Although we compare two large cohorts with ARDS from either COVID-19 or non-COVID-19, analysis was conducted on aggregated data rather than on individual patients due to the difficulty that exists in data sharing from a legislative perspective.

Another limitation is that we excluded patients with a history of chronic kidney disease at baseline, which is a risk factor for the development of AKI [5], although this has not been noted in all COVID-19 cohort studies [39, 40]. We included an analysis of the effects of including patients with CKD which did not change the findings of the study. Additionally, we also used only a serum creatinine-based definition of AKI as reporting of urine output was not reliably recorded. In a multicentre study on AKI incidence from ICU’s in Belgium, the use of both serum creatinine and urine output-based definition markedly increased the incidence of AKI but was consistent with our study when serum creatinine-based measure was used. In this study, when urine output-based definition of AKI was used, only stage 3 AKI was associated with mortality [39].

## Conclusion

AKI is a common and serious complication of COVID-19, and understanding differences in its development provides clues as to its underlying pathophysiology. Compared to a non-COVID-19 ARDS cohort requiring IMV, the incidence of early AKI was lower, but with a higher mortality rate. Geo-economic differences exist in the risk and mortality rates in association with AKI, which may be related to resource availability or underlying genetic susceptibility and requires further study. Important differences exist in the profile of AKI in COVID-19 versus non-COVID-19 ARDS in terms of their haemodynamic profile, time of onset and clinical outcomes.

## Supplementary Information

**Additional file 1: Fig. S1.** Flow chart for outcome analysis for LUNG-SAFE study. *AKI* acute kidney injury; *CKD* chronic kidney disease; *MV* mechanical ventilation. **Fig. S2.** Flow chart for outcome analysis including chronic kidney disease (CKD). *AKI* acute kidney injury; *CKD* chronic kidney disease; *MV* mechanical ventilation. **Fig. S3.** Kaplan–Meier plot of 28-day hospital survival and AKI stage (including CKD). **Fig. S4.** Kaplan–Meier plot of 90-day hospital survival and AKI stage (including CKD). **Fig. S5.** Hazard ratio plots for 28-day ICU mortality against AKI stage (including CKD patients). **Fig. S6.** Hazard ratio plots for 90-day hospital mortality against AKI stage (including CKD patients). **Fig. S7.** Number of missing observations per variable included in analysis. **Fig. S8.** Hazard ratio plots for 28-day ICU mortality against AKI stage (using geographic region). **Fig. S9.** Hazard ratio plots for 90-day hospital mortality against AKI stage (using geographic region).

**Additional file 2: Table S1.** Time frame of development of AKI in patients with COVID-19 versus non-COVID-19 ARDS. **Table S2.** Patients with elevated SOFA cardiovascular scores in patients with COVID-19 versus non-COVID-19 ARDS. **Table S3.** Outcomes of patients with non-COVID-19 ARDS stratified by the presence of AKI. **Table S4.** Cox proportional hazards model of 28-day mortality. **Table S5.** Cox proportional hazards model of 90-day mortality in hospital. **Table S6.** Cox proportional hazards model of 28-day mortality on invasive mechanical ventilation using 412 patients with fully observed data (complete cases). **Table S7.** Cox proportional hazards model of 90-day mortality in hospital using 412 patients with fully observed data (complete cases). **Table S8.** Incidence of acute kidney injury during each 3-month period for the CCCC study.

## Data Availability

The data sets used and/or analysed during the current study are available from the corresponding author upon reasonable request.
