# Supplementary material for: Epidemiology and outcomes of early-onset AKI in COVID-19-related ARDS in comparison with non-COVID-19-related ARDS: insights from two prospective global cohort studies
Source: Crit Care. 2023 Jan 5;27:3. doi: 10.1186/s13054-022-04294-5 (PMC9814373; doi:10.1186/s13054-022-04294-5)
Supplement: Supplementary file 1 — Additional file 1: Fig. S1. Flow chart for outcome analysis for LUNG-SAFE study. AKI acute kidney injury; CKD chronic kidney disease; MV mechanical ventilation. Fig. S2. Flow chart for outcome analysis including chronic kidney disease (CKD). AKI acute kidney injury; CKD chronic kidney disease; MV mechanical ventilation. Fig. S3. Kaplan–Meier plot of 28-day hospital survival and AKI stage (including CKD). Fig. S4. Kaplan–Meier plot of 90-day hospital survival and AKI stage (including CKD). Fig. S5. Hazard ratio plots for 28-day ICU mortality against AKI stage (including CKD patients). Fig. S6. Hazard ratio plots for 90-day hospital mortality against AKI stage (including CKD patients). Fig. S7. Number of missing observations per variable included in analysis. Fig. S8. Hazard ratio plots for 28-day ICU mortality against AKI stage (using geographic region). Fig. S9. Hazard ratio plots for 90-day hospital mortality against AKI stage (using geographic region). [file 13054_2022_4294_MOESM1_ESM.pptx]

## Slide 1
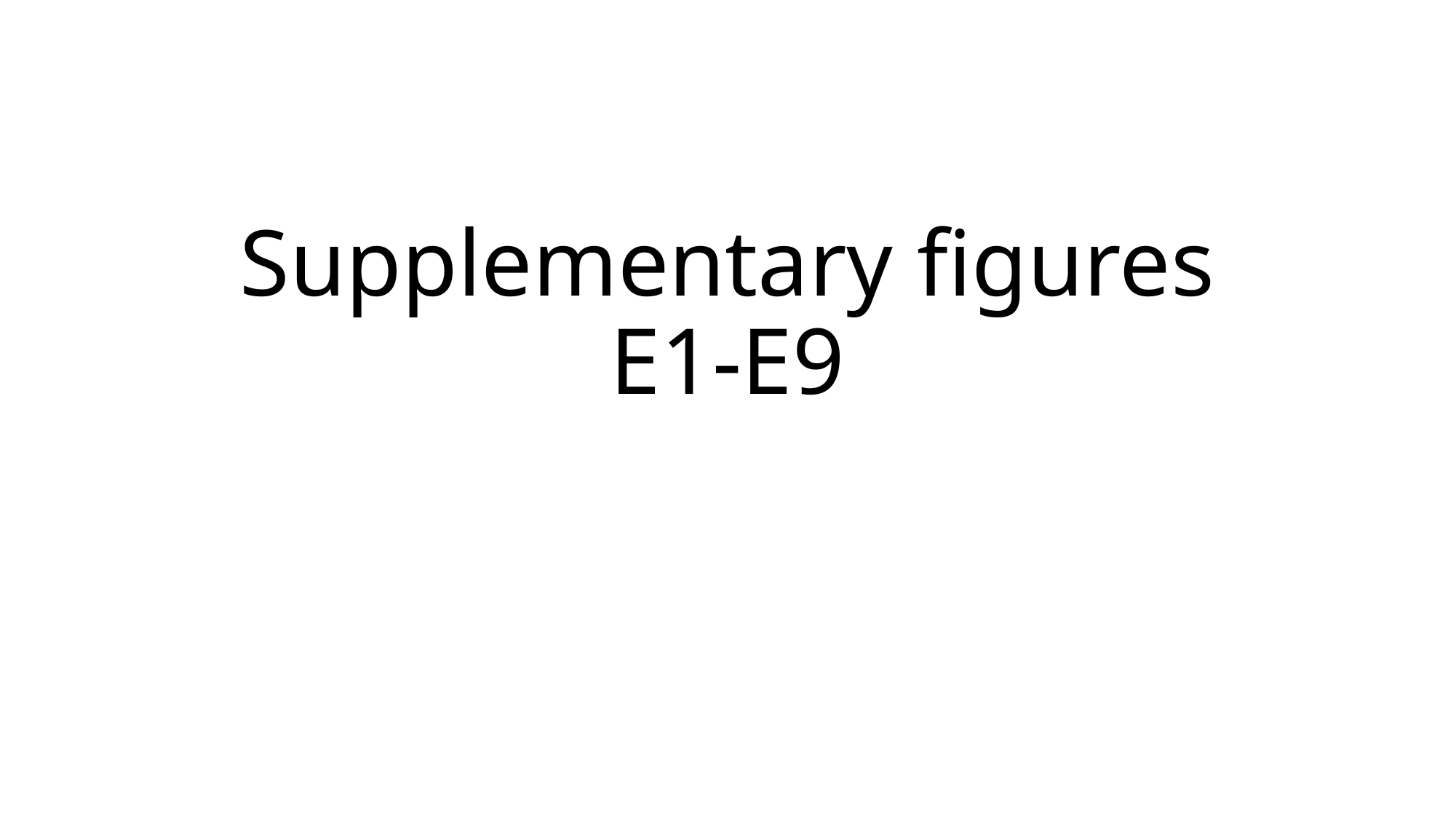

# Supplementary figures E1-E9

## Slide 2
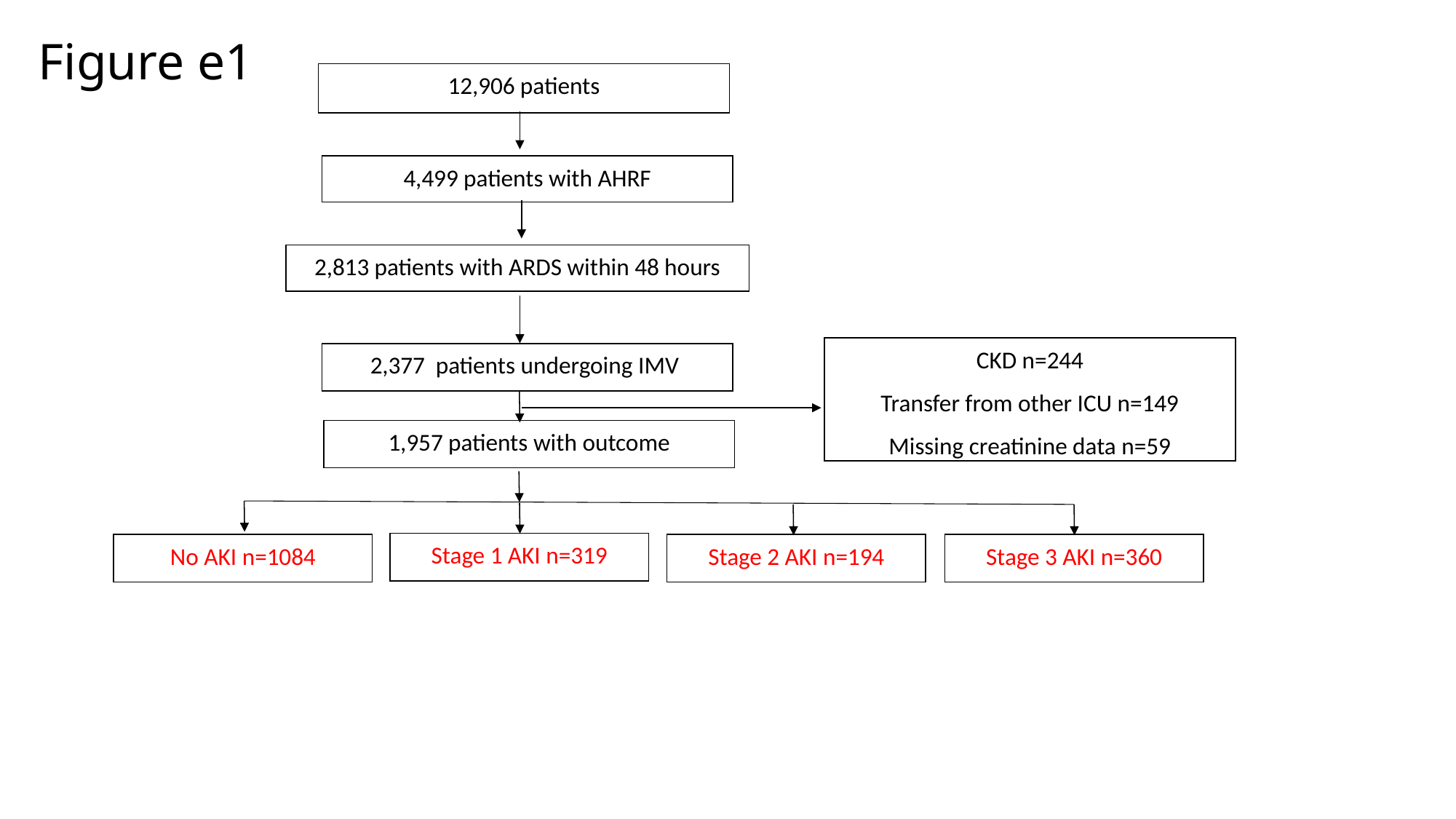

# Figure e1
12,906 patients
4,499 patients with AHRF
2,813 patients with ARDS within 48 hours
CKD n=244
Transfer from other ICU n=149
Missing creatinine data n=59
2,377 patients undergoing IMV
No AKI n=1084
1,957 patients with outcome
Stage 1 AKI n=319
Stage 2 AKI n=194
Stage 3 AKI n=360

## Slide 3
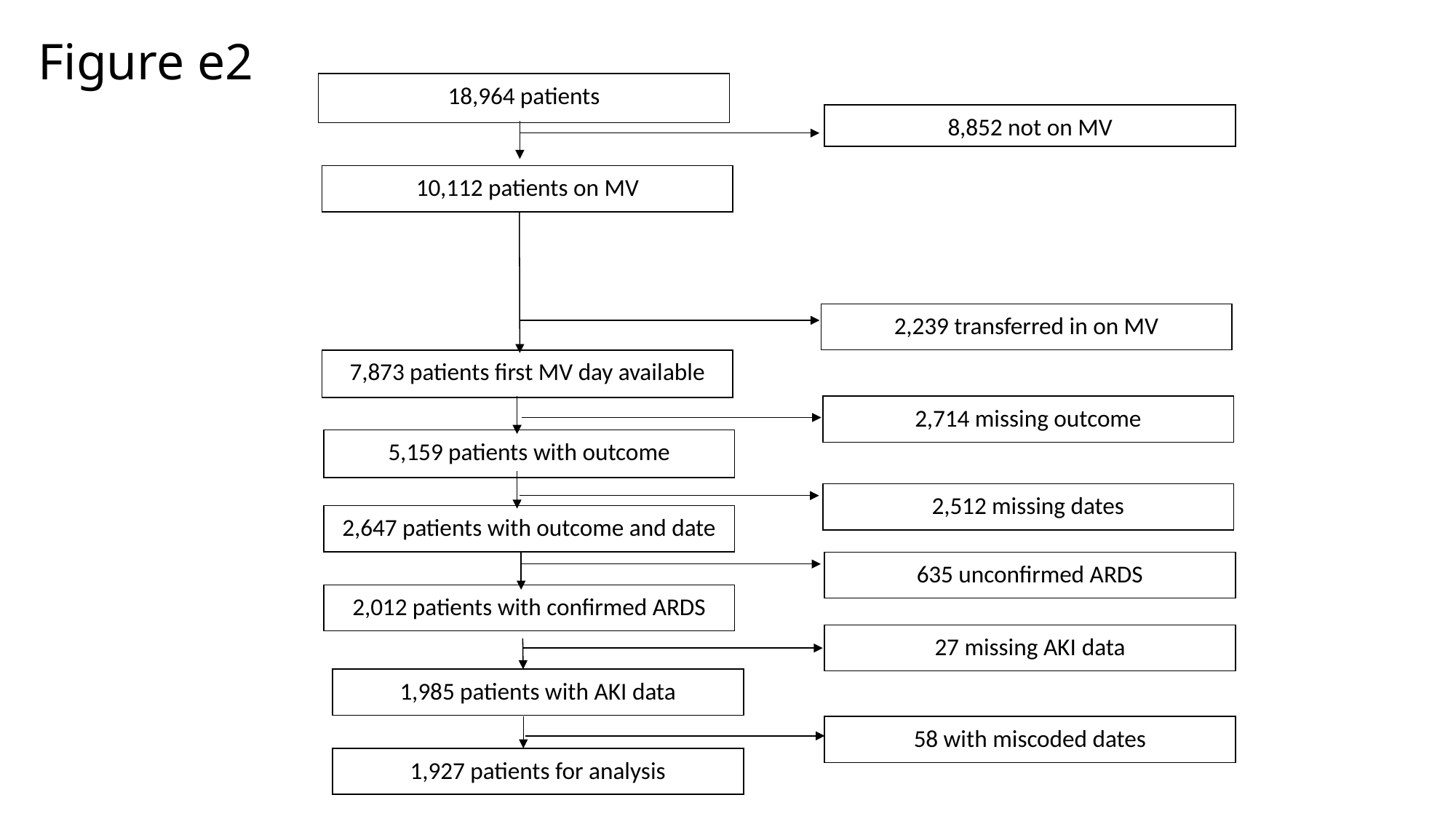

Figure e2
18,964 patients
8,852 not on MV
10,112 patients on MV
2,239 transferred in on MV
7,873 patients first MV day available
2,714 missing outcome
2,512 missing dates
2,647 patients with outcome and date
635 unconfirmed ARDS
2,012 patients with confirmed ARDS
27 missing AKI data
1,985 patients with AKI data
58 with miscoded dates
1,927 patients for analysis
5,159 patients with outcome

## Slide 4
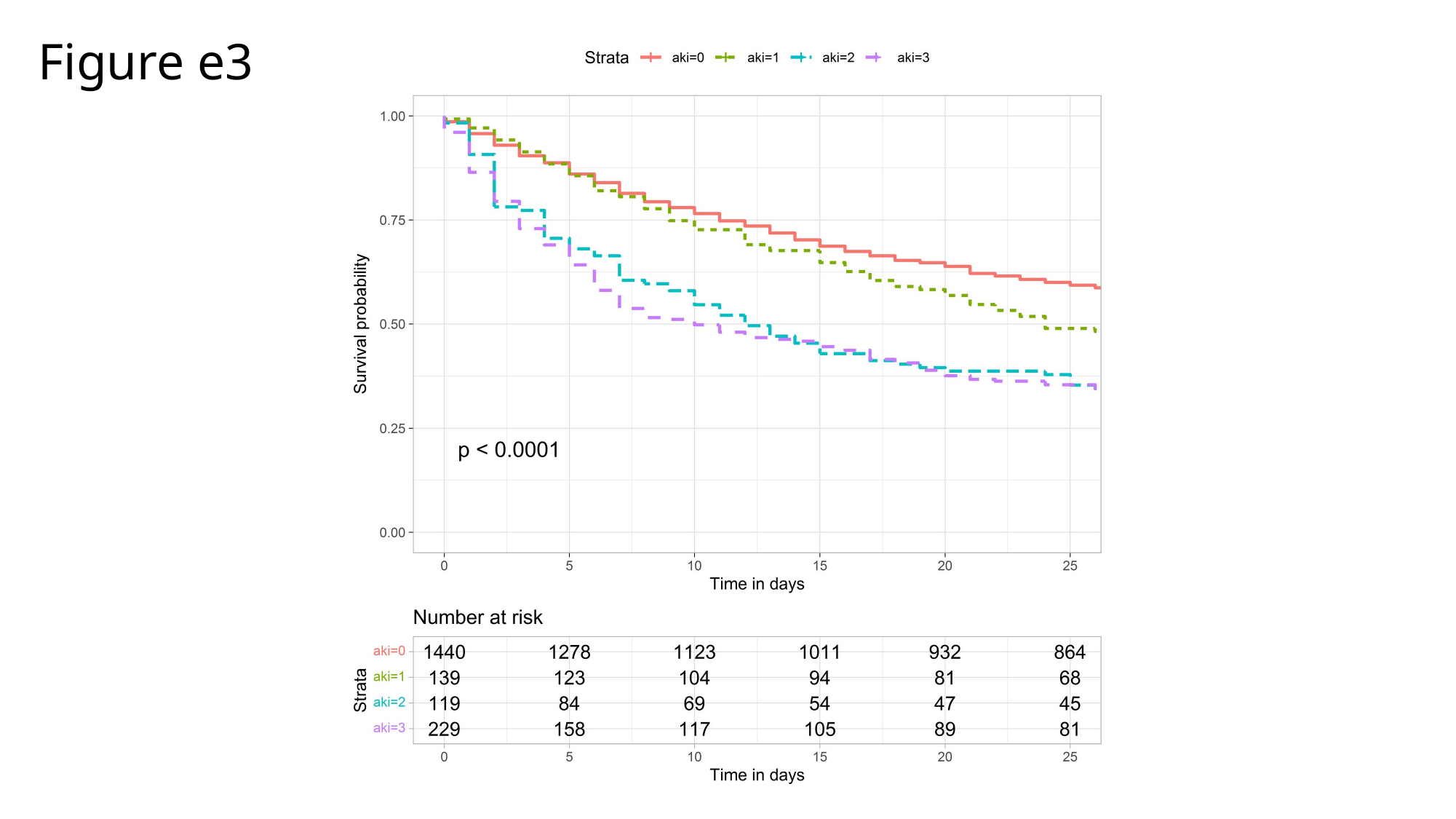

Figure e3

## Slide 5
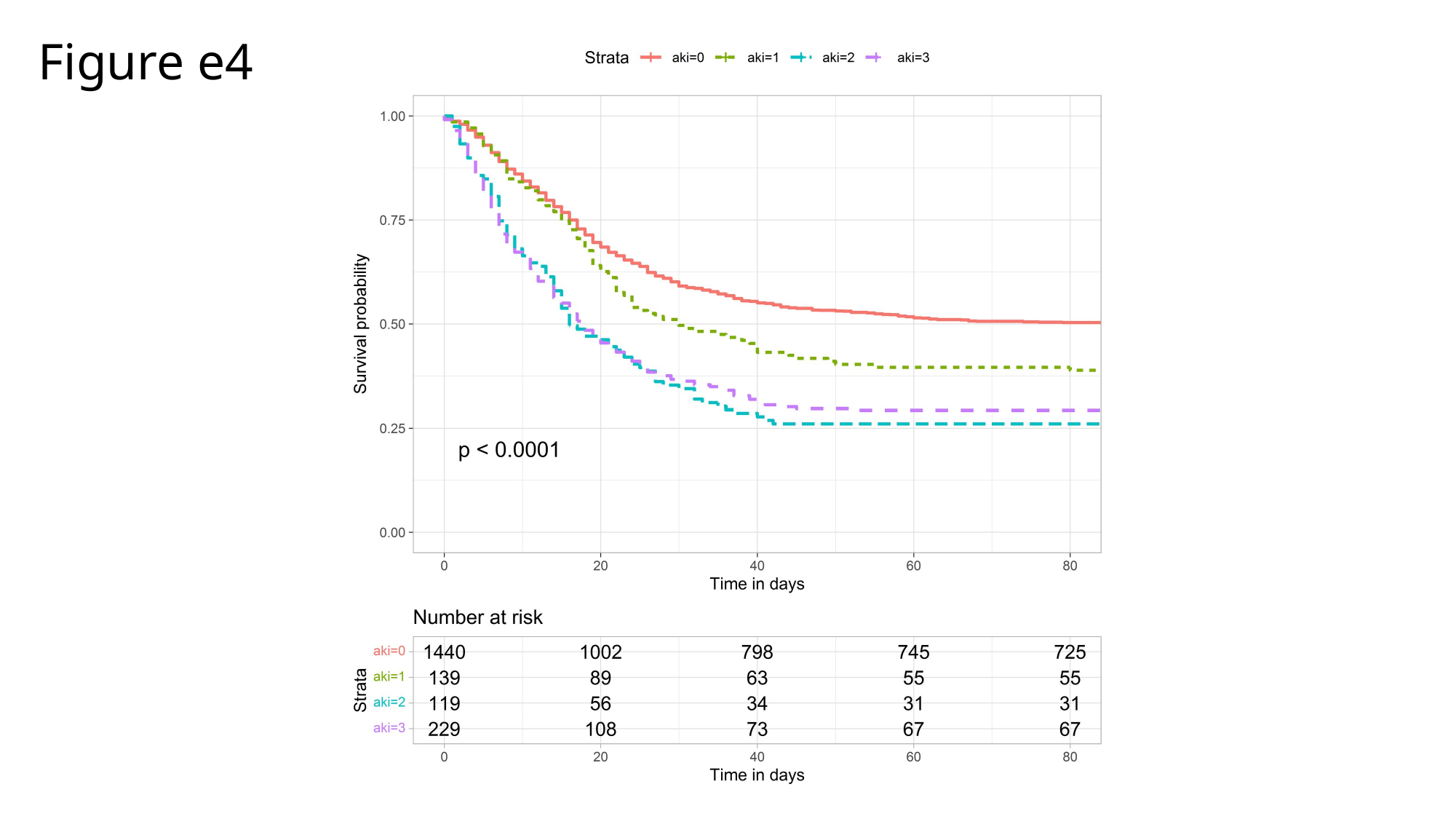

Figure e4

## Slide 6
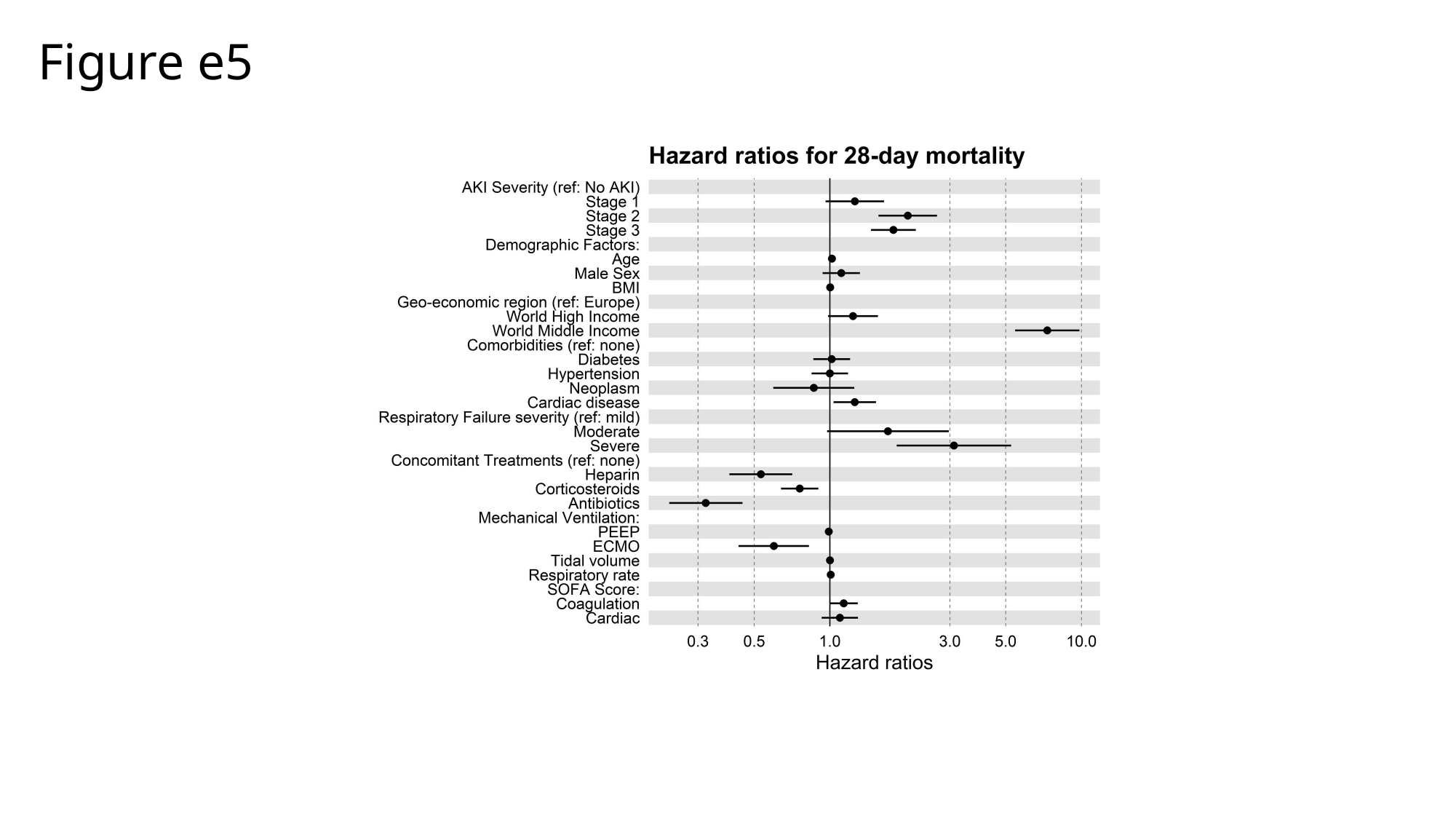

Figure e5

## Slide 7
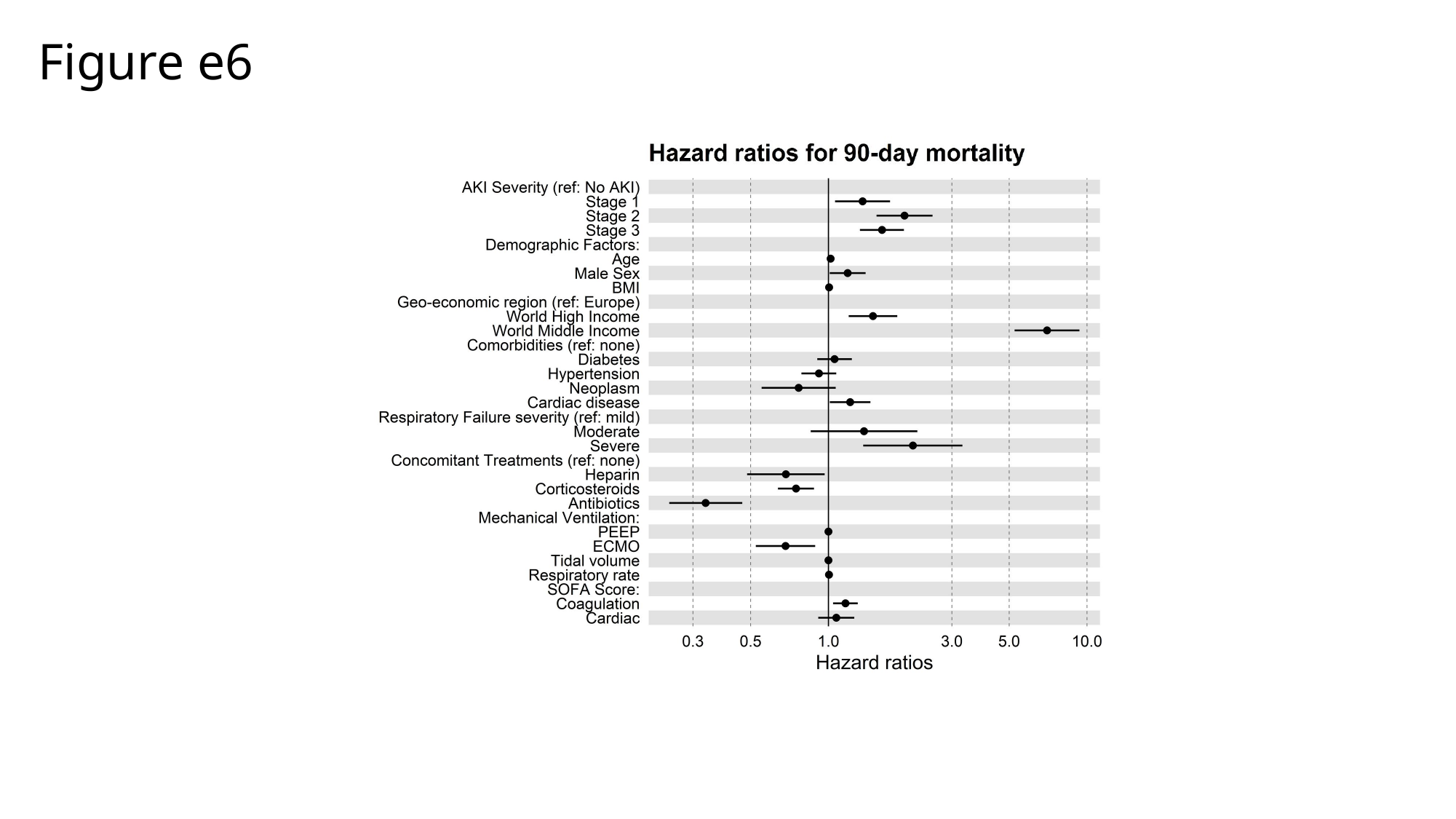

Figure e6

## Slide 8
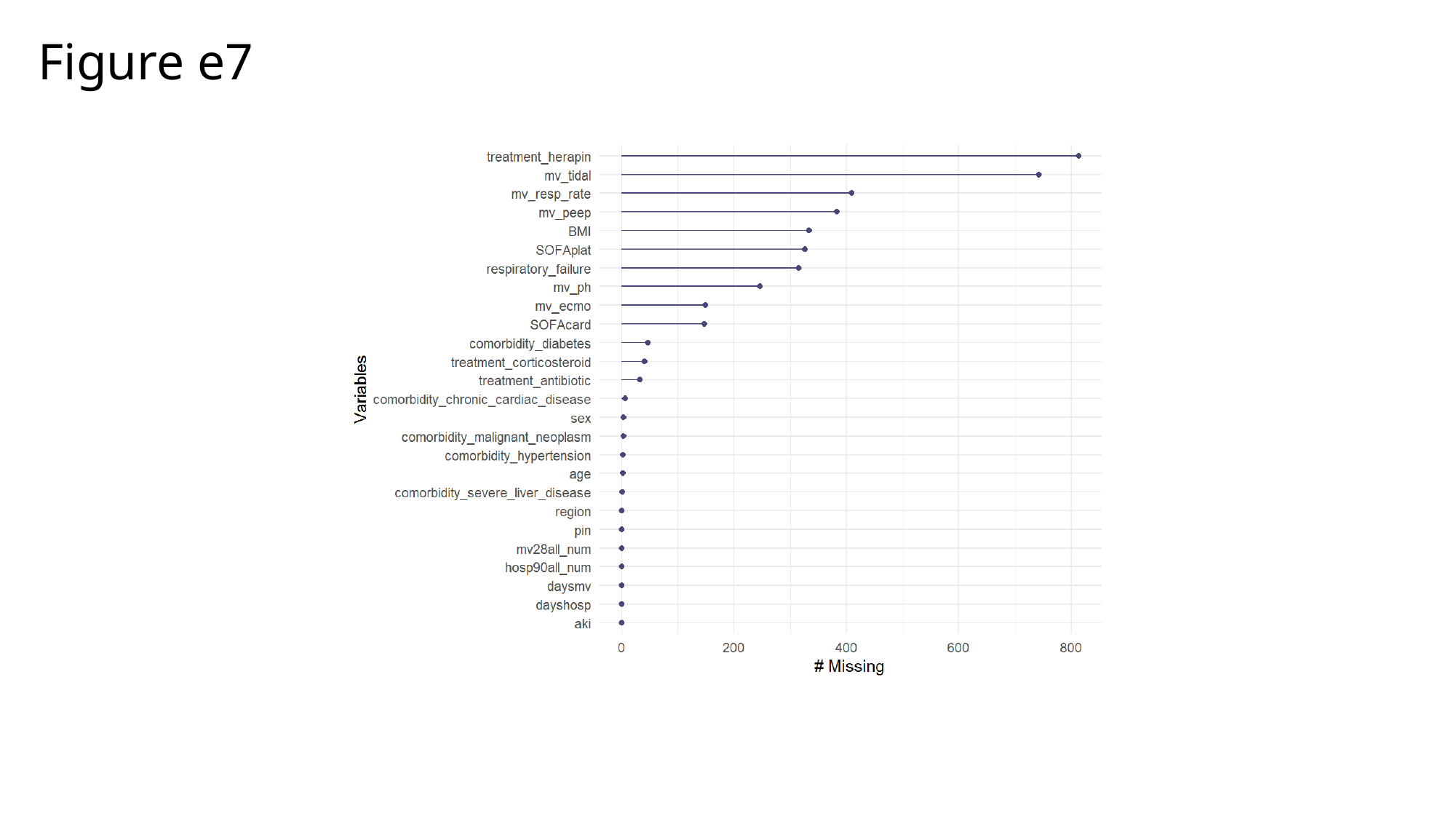

Figure e7

## Slide 9
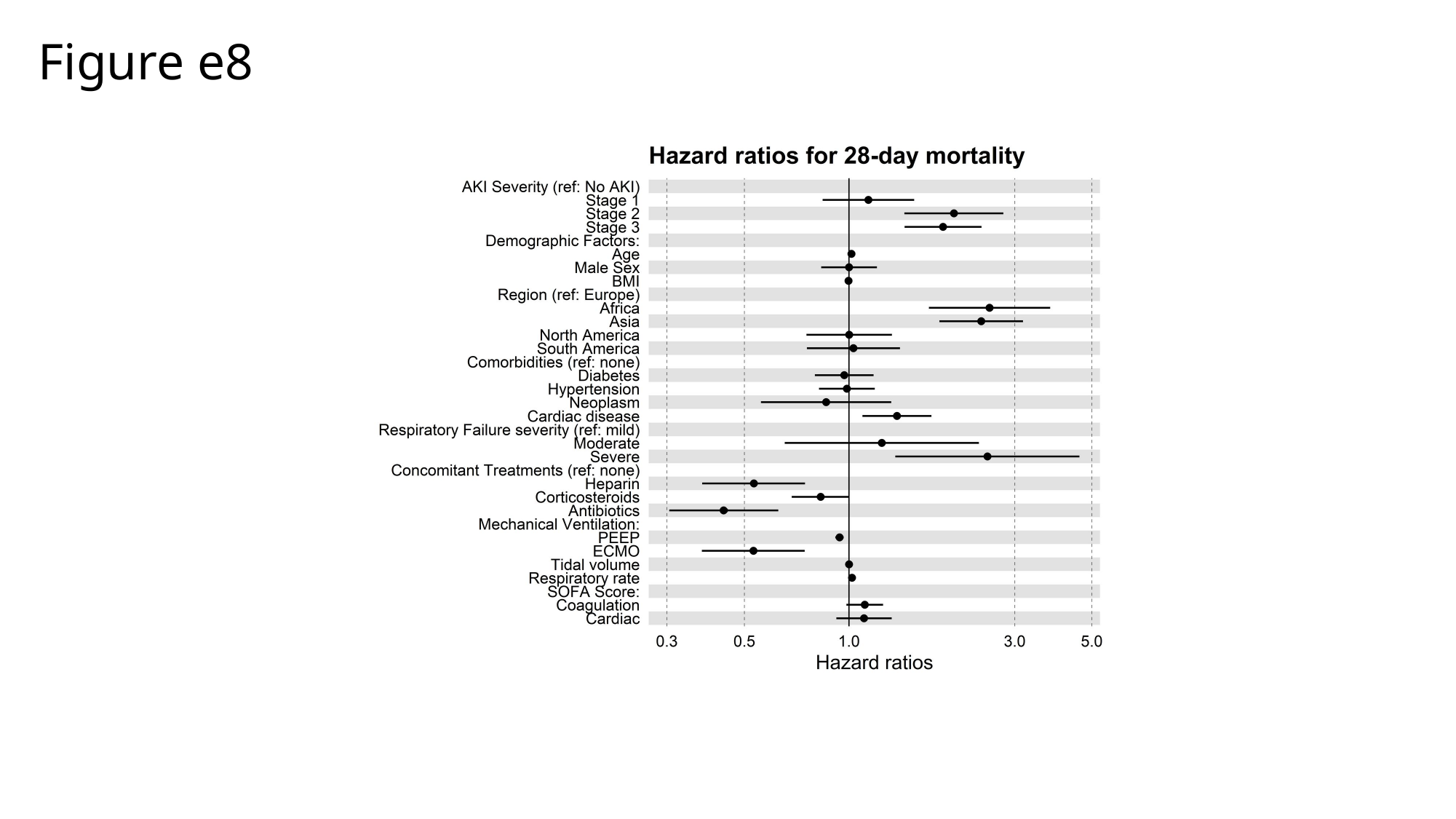

Figure e8

## Slide 10
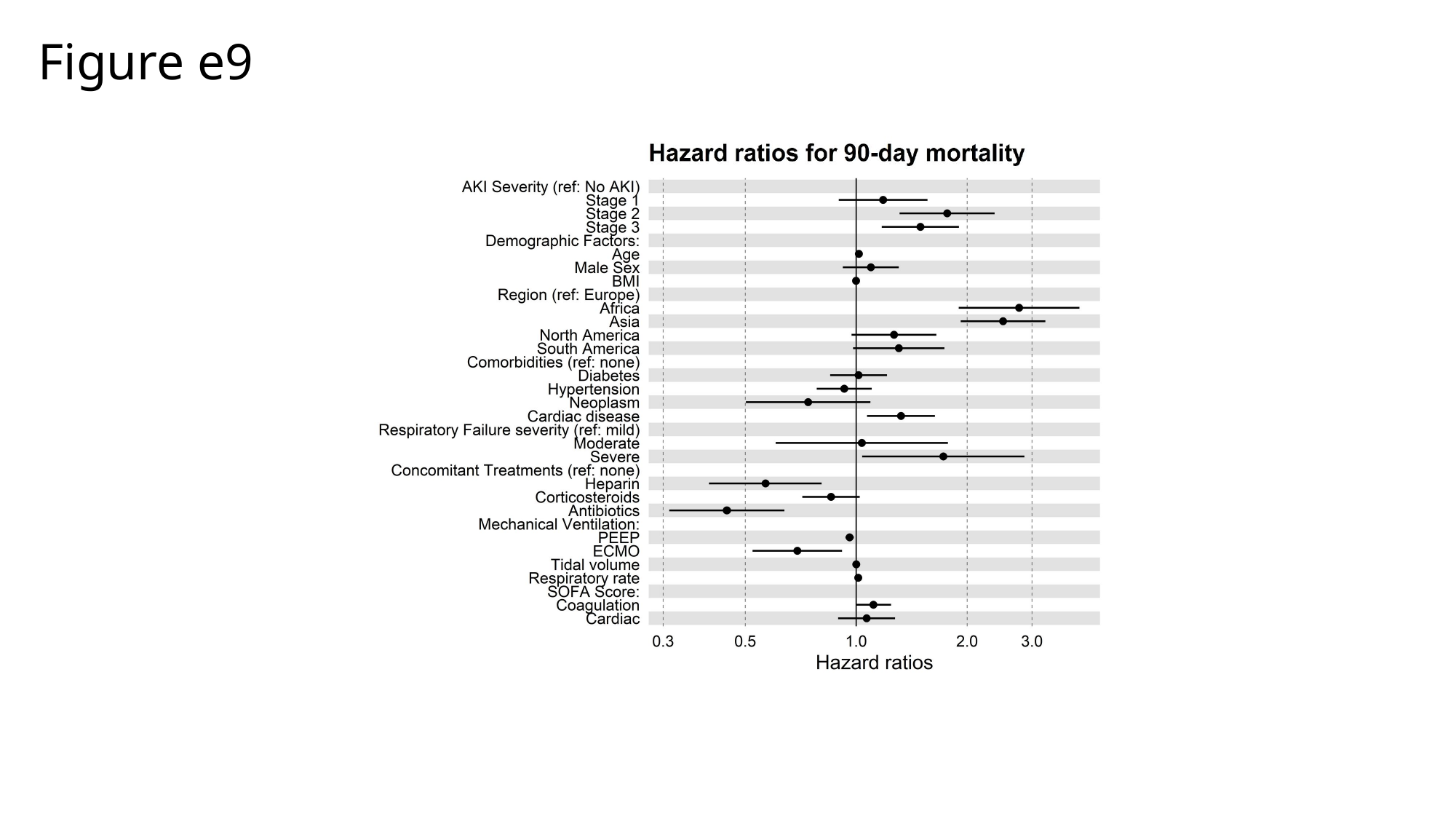

Figure e9
