# Supplementary material for: Epidemiology and outcomes of early-onset AKI in COVID-19-related ARDS in comparison with non-COVID-19-related ARDS: insights from two prospective global cohort studies
Source: Crit Care. 2023 Jan 5;27:3. doi: 10.1186/s13054-022-04294-5 (PMC9814373; doi:10.1186/s13054-022-04294-5)
Supplement: Supplementary file 2 — Additional file 2: Table S1. Time frame of development of AKI in patients with COVID-19 versus non-COVID-19 ARDS. Table S2. Patients with elevated SOFA cardiovascular scores in patients with COVID-19 versus non-COVID-19 ARDS. Table S3. Outcomes of patients with non-COVID-19 ARDS stratified by the presence of AKI. Table S4. Cox proportional hazards model of 28-day mortality. Table S5. Cox proportional hazards model of 90-day mortality in hospital. Table S6. Cox proportional hazards model of 28-day mortality on invasive mechanical ventilation using 412 patients with fully observed data (complete cases). Table S7. Cox proportional hazards model of 90-day mortality in hospital using 412 patients with fully observed data (complete cases). Table S8. Incidence of acute kidney injury during each 3-month period for the CCCC study. [file 13054_2022_4294_MOESM2_ESM.docx]

**Table e1: Timeframe of development of AKI in patients with COVID versus non-COVID ARDS**

|  | **COVID ARDS CCCC Cohort**  **(n= 1,699)**^1^ | **Non-COVID ARDS LUNG SAFE cohort**  **(n= 1,957)**^1^ | **p-value^2^** |
| --- | --- | --- | --- |
| First 2 days of Invasive MV | 355 (21.3%) | 873 (44.6%) | <0.001 |
| Days 3 – 7 of Invasive MV | 149 (8.9%) | 67 (3.4%) | <0.001 |
| Days 8 – 14 | 73 (4.4%) | 29 (1.5%) | <0.001 |
| Days 15 - 21 | 41 (2.4%) | 6 (0.3%) | <0.001 |
| Days 22 - 28 | 11 (0.7%) | 1 (0.1%) | 0.004 |
| Total patients | 629 (37.7%) | 976 (49.9%) | <0.001 |

**Notes**:

1. n (%)

2. Independent samples proportion test

**Table e2 Patients with elevated SOFA Cardiovascular scores in patients with COVID versus non-COVID ARDS**

|  | **COVID ARDS CCCC Cohort**  **(n= 1,699)** ^1^ | **Non-COVID ARDS LUNG SAFE cohort**  **(n= 1,947)** ^1^ | **p-value^2^** |
| --- | --- | --- | --- |
| No AKI | 215 (16%) | 770 (71%) | <0.001 |
| AKI Stage 1 | 27 (25%) | 262 (82%) | <0.001 |
| AKI Stage 2 | 29 (31%) | 178 (92%) | <0.001 |
| AKI Stage 3 | 35 (23%) | 324 (90%) | <0.001 |

**Notes**:

1. n (%); Mean (SD)

2. Independent samples proportion test

**Table e3: Outcomes of patients with non-COVID ARDS stratified by presence of AKI**

|  | **n** | **No AKI**^1^ | **Stage 1**  **AKI^1^** | **Stage 2**  **AKI^1^** | **Stage 3**  **AKI^1^** | **p-value^2^** |
| --- | --- | --- | --- | --- | --- | --- |
| Duration of invasive mechanical ventilation, median (IQR), days   - All - Survivors | 1866  1189 | 12 (14)  12 (14) | 12 (13)  12 (14) | 11 (12)  11 (8) | 13 (15)  16 (17) | 0.184  <0.001 |
| Days in ICU   - All - Survivors | 1957  1280 | 15 (15)  16 (16) | 14 (15)  16 (16) | 12 (12)  14 (11) | 16 (17)  21 (19) | 0.002  <0.001 |
| Days in Hospital   - All - Survivors | 1914  1146 | 25 (21)  28 (22) | 24 (24)  31 (26) | 20 (22)  33 (26) | 23 (24)  39 (27) | <0.001  <0.001 |

**Notes**:

1. n (%); Mean (SD)

2. Pearson's Chi-squared test (categorical); independent sample t-test (continuous)

| **Table e4 Cox proportional hazards model of 28-day mortality** | | | | |
| --- | --- | --- | --- | --- |
| **Variable** | **Hazard Ratio** | **lower .95** | **upper .95** | **p-value** |
| Stage 1 AKI | 1.195 | 0.882 | 1.619 | 0.249 |
| Stage 2 AKI | 2.395 | 1.767 | 3.247 | <0.001 |
| Stage 3 AKI | 2.082 | 1.636 | 2.65 | <0.001 |
| Age (years) | 1.023 | 1.016 | 1.029 | <0.001 |
| Sex (male) | 1.032 | 0.850 | 1.254 | 0.748 |
| Body mass index (kg/m^2^) | 1.002 | 0.987 | 1.017 | 0.786 |
| Region (World High income vs Europe) | 1.187 | 0.922 | 1.527 | 0.183 |
| Region (World Middle income vs Europe) | 7.548 | 5.513 | 10.332 | <0.001 |
| Comorbidity  Diabetes | 0.990 | 0.821 | 1.193 | 0.913 |
| Hypertension | 0.951 | 0.79 | 1.145 | 0.595 |
| Malignant neoplasm | 0.842 | 0.547 | 1.296 | 0.433 |
| Cardiac disease | 1.277 | 1.017 | 1.604 | 0.036 |
| Respiratory failure (moderate vs mild) | 1.548 | 0.812 | 2.951 | 0.184 |
| Respiratory failure (severe vs mild) | 2.935 | 1.59 | 5.417 | 0.001 |
| Heparin | 0.522 | 0.321 | 0.850 | 0.012 |
| Corticosteroid | 0.740 | 0.608 | 0.901 | 0.003 |
| Antibiotic | 0.321 | 0.222 | 0.464 | <0.001 |
| Positive end-expiratory pressure (PEEP, cmH2O) | 0.988 | 0.959 | 1.019 | 0.405 |
| Extracorporeal membrane oxygenation (ECMO) Support | 0.571 | 0.405 | 0.803 | 0.001 |
| Tidal Volume (mL) | 1.000 | 0.999 | 1.002 | 0.45 |
| Respiratory Rate (breaths per minute) | 1.015 | 1.002 | 1.028 | 0.029 |
| Sequential Organ Failure Assessment (Platelets) | 1.189 | 1.051 | 1.345 | 0.006 |
| Sequential Organ Failure Assessment (Cardiovascular) | 1.086 | 0.912 | 1.292 | 0.353 |

| **Table e5 Cox proportional hazards model of 90-day mortality in hospital** | | | | |
| --- | --- | --- | --- | --- |
| **Variable** | **Hazard Ratio** | **lower .95** | **upper .95** | **p-value** |
| Stage 1 AKI | 1.232 | 0.937 | 1.619 | 0.135 |
| Stage 2 AKI | 2.067 | 1.553 | 2.752 | <0.001 |
| Stage 3 AKI | 1.596 | 1.255 | 2.029 | <0.001 |
| Age (years) | 1.022 | 1.015 | 1.029 | <0.001 |
| Sex (male) | 1.132 | 0.944 | 1.357 | 0.181 |
| Body mass index (kg/m^2^) | 1.004 | 0.989 | 1.018 | 0.614 |
| Region (World High income vs Europe) | 1.455 | 1.146 | 1.846 | 0.002 |
| Region (World Middle income vs Europe) | 7.216 | 5.306 | 9.813 | <0.001 |
| Comorbidity  Diabetes | 1.052 | 0.881 | 1.255 | 0.574 |
| Hypertension | 0.893 | 0.752 | 1.059 | 0.193 |
| Malignant neoplasm | 0.690 | 0.465 | 1.024 | 0.065 |
| Cardiac disease | 1.215 | 0.979 | 1.507 | 0.077 |
| Respiratory failure (moderate vs mild) | 1.309 | 0.762 | 2.25 | 0.329 |
| Respiratory failure (severe vs mild) | 2.001 | 1.201 | 3.335 | 0.008 |
| Heparin | 0.574 | 0.346 | 0.952 | 0.034 |
| Corticosteroid | 0.765 | 0.64 | 0.914 | 0.003 |
| Antibiotic | 0.334 | 0.231 | 0.484 | <0.001 |
| Positive end-expiratory pressure (PEEP, cmH2O) | 1.001 | 0.973 | 1.028 | 0.997 |
| Extracorporeal membrane oxygenation (ECMO) Support | 0.697 | 0.529 | 0.919 | 0.011 |
| Tidal Volume (mL) | 1.000 | 0.999 | 1.001 | 0.586 |
| Respiratory Rate (breaths per minute) | 1.007 | 0.996 | 1.019 | 0.192 |
| Sequential Organ Failure Assessment (Platelets) | 1.190 | 1.042 | 1.358 | 0.011 |
| Sequential Organ Failure Assessment (Cardiovascular) | 1.076 | 0.908 | 1.274 | 0.398 |

| **Table e6 Cox proportional hazards model of 28-day mortality on invasive mechanical ventilation using 412 patients with fully observed data (complete cases)** | | | | |
| --- | --- | --- | --- | --- |
| **Variable** | **Hazard Ratio** | **lower .95** | **upper .95** | **p-value** |
| Stage 1 AKI | 0.583 | 0.300 | 1.136 | 0.113 |
| Stage 2 AKI | 1.407 | 0.752 | 2.632 | 0.286 |
| Stage 3 AKI | 1.680 | 0.986 | 2.863 | 0.056 |
| Age (years) | 1.038 | 1.023 | 1.053 | <0.001 |
| Sex (male) | 1.273 | 0.858 | 1.889 | 0.231 |
| Body mass index (kg/m^2^) | 1.011 | 0.985 | 1.038 | 0.398 |
| Region (World High income vs Europe) | 1.224 | 0.762 | 1.965 | 0.403 |
| Region (World Middle income vs Europe) | 32.42 | 16.26 | 64.65 | <0.001 |
| Comorbidity  Diabetes | 0.734 | 0.504 | 1.068 | 0.106 |
| Hypertension | 0.878 | 0.608 | 1.267 | 0.486 |
| Malignant neoplasm | 0.525 | 0.209 | 1.319 | 0.17 |
| Cardiac disease | 1.058 | 0.683 | 1.640 | 0.801 |
| Respiratory failure (moderate vs mild) | 0.878 | 0.292 | 2.644 | 0.817 |
| Respiratory failure (severe vs mild) | 1.430 | 0.503 | 4.064 | 0.502 |
| Heparin | 0.270 | 0.161 | 0.453 | <0.001 |
| Corticosteroid | 0.691 | 0.485 | 0.985 | 0.041 |
| Antibiotic | 0.136 | 0.061 | 0.302 | <0.001 |
| Positive end-expiratory pressure (PEEP, cmH2O) | 1.042 | 0.985 | 1.103 | 0.149 |
| Extracorporeal membrane oxygenation (ECMO) Support | 0.545 | 0.219 | 1.357 | 0.192 |
| Tidal Volume (mL) | 1.000 | 0.998 | 1.002 | 0.700 |
| Respiratory Rate (breaths per minute) | 1.021 | 0.997 | 1.045 | 0.087 |
| Sequential Organ Failure Assessment (Platelets) | 1.451 | 1.113 | 1.891 | 0.006 |
| Sequential Organ Failure Assessment (Cardiovascular) | 1.365 | 0.948 | 1.965 | 0.094 |

| **Table e7 Cox proportional hazards model of 90-day mortality in hospital using 412 patients with fully observed data (complete cases)** | | | | |
| --- | --- | --- | --- | --- |
| **Variable** | **Hazard Ratio** | **lower .95** | **upper .95** | **p-value** |
| Stage 1 AKI | 0.559 | 0.305 | 1.024 | 0.060 |
| Stage 2 AKI | 1.156 | 0.629 | 2.122 | 0.641 |
| Stage 3 AKI | 1.132 | 0.68 | 1.884 | 0.634 |
| Age (years) | 1.031 | 1.017 | 1.045 | <0.001 |
| Sex (male) | 1.618 | 1.126 | 2.325 | 0.009 |
| Body mass index (kg/m^2^) | 1.017 | 0.994 | 1.041 | 0.154 |
| Region (World High income vs Europe) | 1.516 | 0.985 | 2.333 | 0.058 |
| Region (World Middle income vs Europe) | 25.66 | 13.23 | 49.78 | <0.001 |
| Comorbidity  Diabetes | 0.890 | 0.630 | 1.256 | 0.507 |
| Hypertension | 0.790 | 0.558 | 1.118 | 0.183 |
| Malignant neoplasm | 0.493 | 0.223 | 1.089 | 0.08 |
| Cardiac disease | 1.175 | 0.777 | 1.776 | 0.446 |
| Respiratory failure (moderate vs mild) | 0.963 | 0.362 | 2.564 | 0.94 |
| Respiratory failure (severe vs mild) | 1.200 | 0.469 | 3.070 | 0.703 |
| Heparin | 0.332 | 0.201 | 0.548 | <0.001 |
| Corticosteroid | 0.703 | 0.506 | 0.975 | 0.034 |
| Antibiotic | 0.225 | 0.105 | 0.483 | <0.001 |
| Positive end-expiratory pressure (PEEP, cmH2O) | 1.032 | 0.98 | 1.087 | 0.234 |
| Extracorporeal membrane oxygenation (ECMO) Support | 0.491 | 0.221 | 1.094 | 0.082 |
| Tidal Volume (mL) | 0.999 | 0.997 | 1.001 | 0.326 |
| Respiratory Rate (breaths per minute) | 1.024 | 1.002 | 1.046 | 0.031 |
| Sequential Organ Failure Assessment (Platelets) | 1.356 | 1.063 | 1.729 | 0.014 |
| Sequential Organ Failure Assessment (Cardiovascular) | 1.518 | 1.081 | 2.131 | 0.016 |

**Table e8 Incidence of Acute kidney injury during each three month period for the CCCC study**

| **Time-period** | **Acute kidney injury (% of included patients in that period)** |
| --- | --- |
| January to March 2020 | 40 (19) |
| April to June 2020 | 128 (23) |
| July to September 2020 | 63 (19) |
| October to December 2020 | 36 (16) |
| January to March 2021 | 33 (22) |
| April to June 2021 | 38 (27) |
| July to September 2021 | 10 (14) |
| October to December 2021 | 3 (18) |
| January to March 2022 | 4 (40) |
| April to June 2022 | 0 (0) |
