# Supplementary material for: Correction : Epidemiology and outcomes of early-onset AKI in COVID-19-related ARDS in comparison with non-COVID-19-related ARDS: insights from two prospective global cohort studies
Source: Crit Care. 2023 May 26;27:202. doi: 10.1186/s13054-023-04487-6 (PMC10214330; doi:10.1186/s13054-023-04487-6)
Supplement: Supplementary file 1 — Additional file 1. List of collaborating authors in the CCCC Consortium. [file 13054_2023_4487_MOESM1_ESM.docx]

1. **Appendix**

**Contributors**

| **Prefix/First Name/Last Name** | **Site Name** |
| --- | --- |
| Tala Al-Dabbous Huda Alfoudri Mohammed Shamsah | Al Adan Hospital |
| Qamrah Alhadad Matly Hanan | Aljufrah Clinical Isolation Center |
| Subbarao Elapavaluru Ashley Berg Christina Horn | Allegheny General Hospital |
| Ahmed Reda Mohamed Elsayed Abdelhalim  Amro Essam Amer  Cinderella Omar Rageh Elnaggar  Ahmed Ayman Hassan  Ali Abdelaziz  Mohamed Abdelhalim  Yehia Samir Shaaban Aly Orabi | Alexandria Main University Hospital |
| Zinah A. Alaraji  Mo'nes R. Muhaisen  Lana Almasri  Dana Mustafa  Shaher Hamdan  Yousef Al-Saba'a  Zaina Dalloul  Mohammed Alkahlout  Hamza Jaber  Osama Aldabbourosama | Alshifa Hospital, Gaza |
| Aliae AR Mohamed Hussein  Zarief Kamel Emad  Sarah Khaled  Nouralsabah Mohamed  Ebtisam Hassanin  Abdelhafeez Hamdi  Abdelrahman Ragab  Mohammed G Azizeldin | Assiut University Hospital |
| Yunis Mayasi | Avera McKennan Hospital & University Health Centre |
| Stephan Schroll | Barmherzige Bruder Regansburg |
| Dan Meyer Jorge Velazco Ludmyla Ploskanych Wanda Fikes Rohini Bagewadi Marvin Dao Haley White  Alondra Berrios Laviena  Ashley Ehlers Maysoon Shalabi-McGuire Trent Witt | Baylor Scott & White Health |
| Lorenzo Grazioli Luca Lorini | Bergamo Hospital |
| E. Wilson Grandin Jose Nunez Tiago Reyes | Beth Israel Deaconess Medical Centre |
| Diarmuid O’Briain Stephanie Hunter | Box Hill Hospital |
| Mahesh Ramanan Julia Affleck | Caboolture Hospital |
| Hemanth Hurkadli Veerendra  Sumeet Rai Josie Russell-Brown Mary Nourse | Canberra Hospital |
| Mark Joseph Brook Mitchell Martha Tenzer | Carilion Clinic |
| Ryuzo Abe | Chiba University Graduate School of Medicine |
| Hwa Jin Cho In Seok Jeong | Chonnam National University Hospital |
| Nadeem Rahman Vivek Kakar  Helen Sun  Alison Hanley | Cleveland Clinic- Abu Dhabi |
| Nicolas Brozzi | Cleveland Clinic - Florida |
| Omar Mehkri Sudhir Krishnan Abhijit Duggal Stuart Houltham | Cleveland Clinic - Ohio |
| Jerónimo Graf | Clinica Alemana De Santiago |
| Roderigo Diaz Roderigo Orrego  Camila Delgado Joyce González Maria Soledad Sanchez Michael Piagnerelli Josefa Valenzuela Sarrazin | Clinica Las Condez |
| A/Prof. Gustavo Zabert Lucio Espinosa Paulo Delgado Victoria Delgado | Clinica Pasteur National- University of Comahue |
| Diego Fernando Bautista Rincón Angela Maria Marulanda Yanten Melissa Bustamante Duque | Clinica Valle de Lilli |
| Daniel Brodie | Medical ICU, Columbia College of Physicians and Surgeons, New-York-Presbyterian Hospital, NY, NY, USA |
| Khaled Abouelmagd | Dr. Mohammad Alfagih Hospital |
| Alyaa Elhazmi Abdullah Al-Hudaib | Dr Sulaiman Alhabib Medical Group – Research Center, Riyadh, Saudi Arabia |
| Jeff Javidfar  Maria Callahan  Andy Dong  Charles Crepy D'Orleans | Emory University Healthcare System |
| M. Azhari Taufik  Elizabeth Yasmin Wardoyo Margaretha Gunawan Nurindah S Trisnaningrum Vera Irawany Muhammad Rayhan | Fatmawati Hospital |
| Mauro Panigada Antonio Pesenti Alberto Zanella Giacomo Grasselli Sebastiano Colombo  Chiara Martinet  Gaetano Florio | Fondazione IRCCS Policlinico of Milan (Fondazione IRCCS Ca' Granda Ospedale Maggiore Policlinico) |
| Massimo Antonelli Simone Carelli Domenico L. Grieco | Fondazione Policlinico Universitario Agostino Gemelli IRCCS |
| Motohiro Asaki | Fujieda Municipal General Hospital |
| Kota Hoshino | Fukuoka University |
| Leonardo Salazar  Mary Alejandra Mendoza Monsalve | Fundación Cardiovascular de Colombia |
| John Laffey Bairbre McNicholas David Cosgrave  Minha Atif  Fadi Qutishat  Caoimhe Laffey  Michaeala Van Der Walt | Galway University Hospitals |
| Joseph McCaffrey Allison Bone | Geelong Hospital |
| Yusuff Hakeem | Glenfield Hospital |
| James Winearls Mandy Tallott | Gold Coast University Hospital |
| David Thomson  Ivan Joubert Christel Arnold-Day  Jenna Piercy  Richard van Zyl Smit Malcom Miller Lisa Seymour  Francois van Heyningen  Gilbert Teyangesikayi  David Fredericks | Groote Schuur Hospital |
| Ali Ait Hssain Jeffrey Aliudin Al-Reem Alqahtani Khoulod Mohamed Ahmed Mohamed Darwin Tan Joy Villanueva Ahmed Zaqout | Hamad General Hospital - Weill Cornell Medical College in Qatar |
| Ethan Kurtzman Arben Ademi Ana Dobrita Khadija El Aoudi Juliet Segura | Hartford HealthCare |
| Gezy Giwangkancana | Hasan Sadikin Hospital (Adult) |
| Shinichiro Ohshimo | Hiroshima University |
| Javier Osatnik | Hospital Alemán |
| Anne Joosten | Hospital Civil Marie Curie |
| Antoni Torres Minlan Yang Ana Motos | Hospital Clinic, Barcelona |
| Carlos Luna | Hospital de Clínicas |
| Francisco Arancibia | Hospital del Tórax |
| Virginie Williams Alexandre Noel | Hospital du Sacre Coeur (Universite de Montreal) |
| Nestor Luque | Hospital Emergencia Ate Vitarte |
| Marina Fantini | Hospital Mater Dei |
| Ruth Noemi Jorge García Enrique Chicote Alvarez | Hospital Nuestra Señora de Gracia |
| Anna Greti | Hospital Puerta de Hierro |
| Adrian Ceccato | Hospital Universitari Sagrat Cor |
| Angel Sanchez | Hospital Universitario Sant Joan d’Alacant |
| Ana Loza Vazquez | Hospital Universitario Virgen de Valme |
| Ferran Roche-Campo Diego Franch-Llasat | Hospital Verge de la Cinta de Tortosa |
| Divina Tuazon | Houston Methodist Hospital |
| Marcelo Amato Luciana Cassimiro Flavio Pola Francis Ribeiro Guilherme Fonseca | INCOR (Universidade de São Paulo) |
| Heidi Dalton Mehul Desai Erik Osborn Hala Deeb | INOVA Fairfax Hospital |
| Antonio Arcadipane Gennaro Martucci Giovanna Panarello Stefano Vitiello Claudia Bianco Giovanna Occhipinti Matteo Rossetti Raffaele Cuffaro | ISMETT |
| Sung-Min Cho  Glenn Whitman | Johns Hopkins |
| Marwan El Sayed Walaa Mokhtar Eslam El-Shenawy | Kafr Elsheikh University Hospital |
| Hiroaki Shimizu Naoki Moriyama | Kakogawa Acute Care Medical Center |
| Jae-Burm Kim | Keimyung University Dong San Hospital |
| Nobuya Kitamura | Kimitsu Chuo Hospital |
| Johannes Gebauer | Klinikum Passau |
| Toshiki Yokoyama | Kouritu Tousei Hospital |
| Abdulrahman Al-Fares Sarah Buabbas Esam Alamad Fatma Alawadhi Kalthoum Alawadi  Mohamed Ahmed Khalefa  Nourah Ahmad Abdulaziz Al Ajeel  Mohammad Fathy Aly  Abdullah Al-Saleh  Abdullah Naanouh  Alaa Mohammed Elshourbgy  Abdulrahman Al-Fares  Mohamed Yousef Gad  Rania Mohamed ElRazaz  Ibrahim Khadadah  Ahmed Mohammed Almumin  Hala Altarakma  Hasan Albannay  Mohammed Kh Alsaleh  Mahmoud Saad Abdallah Radwan  Islam Ahmed Saadallah | Al-Amiri and Jaber Al-Ahmed Hospitals, Kuwait Extracorporeal Life Support Program |
| Hiro Tanaka | Kyoto Medical Centre |
| Satoru Hashimoto Masaki Yamazaki | Kyoto Prefectural University of Medicine |
| Tak-Hyuck Oh | Kyung Pook National University Chilgok Hospital |
| Mark Epler Cathleen Forney  Louise Kruse Jared Feister Joelle Williamson Katherine Grobengieser | Lancaster General Health |
| Eric Gnall Sasha Golden Mara Caroline  Timothy Shapiro Colleen Karaj Lisa Thome Lynn Sher Mark Vanderland Mary Welch Sherry McDermott | Lankenau Institute of Medical Research (Main Line Health) |
| Matthew Brain Sarah Mineall  Maria Unwin  Lixian Chen  Tarnya Trezise  Laurie McKeon | Launceston General Hospital |
| Dai Kimura | Le Bonheur Children’s Hospital |
| Luca Brazzi Gabriele Sales  Giorgia Montrucchio | Le Molinette Hospital (Ospedale Molinette Torino) |
| Tawnya Ogston | Legacy Emanuel Medical Center |
| Dave Nagpal Karlee Fischer | London Health Sciences Centre |
| Roberto Lorusso  Bas van Bussell  Maria Elena De Piero  Silvia Mariani | Maastricht University Medical Centre |
| Dr Rajavardhan Rangappa Dr Rajesh Mohan Shetty  Sujin Rai P  Argin Ganesan | Manipal Hospital Whitefield |
| Mariano Esperatti  Nora Angélica Fuentes  Maria Eugenia Gonzalez | Hospital Privado de Comunidad. Mar del Plata. Escuela Superior de Medicina. Universidad Nacional de  Mar del Plata |
| Diarmuid O’Briain | Maroondah Hospital |
| Edmund G. Carton | Mater Misericordiae University Hospital |
| Ayan Sen Amanda Palacios Deborah Rainey | Mayo Clinic College of Medicine |
| Gordan Samoukoviv Josie Campisi | McGill University Health Centre |
| Lucia Durham Emily Neumann Cassandra Seefeldt Octavio Falcucci Amanda Emmrich Jennifer Guy Carling Johns Kelly Potzner Catherine Zimmermann Angelia Espinal | Medical College of Wisconsin (Froedtert Hospital) |
| Nina Buchtele Michael Schwameis  Andrea Korhnfehl  Roman Brock  Thomas Staudinger | Medical University of Vienna |
| Stephanie-Susanne Stecher Michaela Barnikel  Sófia Antón  Alexandra Pawlikowski | Medical Department II, LMU Hospital Munich |
| Akram Zaaqoq Lan Anh Galloway Caitlin Merley | MedStar Washington Hospital Centre |
| Alistair Nichol | Monash University |
| Marc Csete Luisa Quesada Isabela Saba | Mount Sinai Medical Centre |
| Daisuke Kasugai Hiroaki Hiraiwa Taku Tanaka | Nagoya University Hospital |
| Eva Marwali Yoel Purnama Santi Rahayu Dewayanti Ardiyan Dafsah Arifa Juzar Debby Siagian | National Cardiovascular Center Harapan Kita, Jakarta, Indonesia |
| Yih-Sharng Chen | National Taiwan University Hospital |
| Mark Ogino | Nemours Alfred I duPont Hospital for Children |
| Prashant Nasa Christina Matthew Nimisha Abdul Majeed | NMC Al Nahda Hospital Dubai |
| Indrek Ratsep Andra-Maris Post Piret Sillaots  Anneli Krund  Merili-Helen Lehiste  Tanel Lepik | North Estonia Medical Centre |
| Frank Manetta Effe Mihelis Iam Claire Sarmiento Mangala Narasimhan Michael Varrone | Northwell Health |
| Mamoru Komats | Obihiro-Kosei General Hospital |
| Julia Garcia-Diaz Catherine Harmon | Ochsner Clinic Foundation |
| S. Veena Satyapriya Amar Bhatt Nahush A. Mokadam Alberto Uribe Alicia Gonzalez Haixia Shi Johnny McKeown Joshua Pasek Juan Fiorda Marco Echeverria | Ohio State University Medical Centre |
| Rita Moreno | Oklahoma Heart Institute |
| Bishoy Zakhary | Oregon Health and Science University Hospital (OHSU) |
| Marco Cavana Alberto Cucino | Ospedale di Arco (Trento Hospital) |
| Giuseppe Foti Marco Giani Benedetta Fumagalli | Ospedale San Gerardo |
| Davide Chiumello Valentina Castagna | Ospedale San Paolo |
| Andrea Dell’Amore Paolo Navalesi | Padua University Hospital (Policlinico of Padova) |
| Hoi-Ping Shum | Pamela Youde Nethersole Eastern Hospital |
| Alain Vuysteke | Papworth Hospitals NHS Foundation Trust |
| Asad Usman Andrew Acker Benjamin Smood Blake Mergler Federico Sertic Madhu Subramanian Alexandra Sperry Nicolas Rizer | Penn Medicine (Hospital of the University of Pennsylvania) |
| Erlina Burhan  Menaldi Rasmin Ernita Akmal Faya Sitompul Navy Lolong Bhat Naivedh | Persahabatan General Hospital |
| Simon Erickson | Perth Children's Hospital |
| Peter Barrett David Dean Julia Daugherty | Piedmont Atlanta Hospital |
| Antonio Loforte | Policlinico di S. Orsola, Università di Bologna |
| Irfan Khan Mohammed Abraar Quraishi Olivia DeSantis | Presbyterian Hospital Services, Albuquerque |
| Dominic So Darshana Kandamby | Princess Margaret Hospital |
| Jose M. Mandei Hans Natanael | Prof Dr R. D. Kandou General Hospital - Paediatric |
| Eka YudhaLantang Anastasia Lantang | Prof Dr R. D R. D. Kandou General Hospital - Adult |
| Surya Oto Wijaya | Dr Sulianti Saroso Hospital |
| Anna Jung | Providence Saint John's Health Centre |
| George Ng Wing Yiu Ng | Queen Elizabeth Hospital, Hong Kong |
| Pauline Yeung Ng  Shu Fang | The University of Hong Kong |
| Alexis Tabah Megan Ratcliffe Maree Duroux | Redcliffe Hospital |
| Ahmed Alajeeli  Ali Tarhabat | Regdalin Hospital |
| Shingo Adachi Shota Nakao | Rinku General Medical Center (and Senshu Trauma and Critical Care Center) |
| Pablo Blanco Ana Prieto Jesús Sánchez | Rio Hortega University Hospital |
| Meghan Nicholson | Rochester General Hospital |
| Warwick Butt Alyssa Serratore Carmel Delzoppo | Royal Children’s Hospital |
| Pierre Janin Elizabeth Yarad | Royal North Shore Hospital |
| Richard Totaro Jennifer Coles | Royal Prince Alfred Hospital |
| Bambang Pujo | RSUD Soetomo |
| Robert Balk Andy Vissing Esha Kapania James Hays Samuel Fox Garrett Yantosh Pavel Mishin | Rush University, Chicago |
| Saptadi Yuliarto Kohar Hari Santoso Susanthy Djajalaksana | Saiful Anwar Malang Hospital (Brawijaya University) (Paediatrics) |
| Arie Zainul Fatoni | Saiful Anwar Malang Hospital (Brawijaya University) (Adult) |
| Masahiro Fukuda | Saiseikai Senri Hospital |
| Keibun Liu | Saiseikai Utsunomiya Hospital |
| Paolo Pelosi Denise Battaglini | San Martino Hospital |
| Juan Fernando Masa Jiménez | San Pedro de Alcantara Hospital |
| Diego Bastos | Sao Camilo Cura D’ars |
| Sérgio Gaião | São João Hospital Centre, Porto |
| Desy Rusmawatiningtyas | Sardjito Hospital (Paediatrics) |
| Young-Jae Cho | Seoul National University Bundang Hospital |
| Su Hwan Lee | Severance Hospital |
| Tatsuya Kawasaki | Shizuoka Children’s Hospital |
| Laveena Munshi | Sinai Health Systems (Mount Sinai Hospital) |
| Pranya Sakiyalak Prompak Nitayavardhana | Siriraj Hospital |
| Mohamed Bashir Elagili  Talat Ahmed Abu Salem | Soug Althulatha Isolation Center |
| Tamara Seitz | Sozialmedizinisches Zentrum Süd – Kaiser-Franz-Josef-Spital |
| Rakesh Arora David Kent | St Boniface Hospital (University of Mannitoba) |
| Daniel Marino | St Christopher’s Hospital for Children |
| Swapnil Parwar Andrew Cheng Jennene Miller | St George Hospital |
| Shigeki Fujitani Naoki Shimizu | St Marianna Medical University Hospital |
| Jai Madhok Clark Owyang | Stanford University Hospital |
| Hergen Buscher Claire Reynolds | St Vincent’s Hospital |
| Olavi Maasikas AleksanBeljantsev Vladislav Mihnovits | Tartu University Hospital |
| Takako Akimoto Mariko Aizawa Kanako Horibe Ryota Onodera | Teine Keijinkai Hospital |
| Carol Hodgson Aidan Burrell Meredith Young | The Alfred Hospital |
| Timothy George | The Heart Hospital Baylor Plano, Plano |
| Kiran Shekar  Niki McGuinness Lacey Irvine | The Prince Charles Hospital |
| Brigid Flynn | The University of Kansas Medical Centre |
| Tomoyuki Endo | Tohoku Medical and Pharmaceutical University |
| Kazuhiro Sugiyama | Tokyo Metropolitan Bokutoh Hospital |
| Keiki Shimizu | Tokyo Metropolitan Medical Center |
| Eddy Fan Kathleen Exconde | Toronto General Hospital |
| Shingo Ichiba | Tokyo Women’s Medical University Hospital |
| Muhannud Binnawara | Tripoli Central Hospital |
| Leslie Lussier | Tufts Medical Centre (and Floating Hospital for Children) |
| Gösta Lotz | Universitätsklinikum Frankfurt (University Hospital Frankfurt) (Uniklinik) |
| Maximilian Malfertheiner Lars Maier Esther Dreier | Universitätsklinikum Regensburg (Klinik für Innere Medizin II) |
| Neurinda Permata Kusumastuti | University Airlangga Hospital (Paediatric) |
| Colin McCloskey Al-Awwab Dabaliz Tarek B Elshazly Josiah Smith | University Hospital Cleveland Medical Centre (UH Cleveland Hospital) |
| Konstanty S. Szuldrzynski Piotr Bielański | University Hospital in Krakow |
| Yusuff Hakeem | University Hospitals of Leicester NHS Trust (Glenfield Hospital) |
| Keith Wille | University of Alabama at Birmingham Hospital (UAB) |
| Srinivas Murthy | University of British Columbia |
| Ken Kuljit S. Parhar Kirsten M. Fiest  Cassidy Codan Anmol Shahid | University of Calgary (Peter Lougheed Centre, Foothills Medical Centre, South Health Campus and Rockyview General Hospital) |
| Mohamed Fayed Timothy Evans Rebekah Garcia Ashley Gutierrez Hiroaki Shimizu | University of California, San Francisco-Fresno Clinical Research Centre |
| Tae Song Rebecca Rose | University of Chicago |
| Suzanne Bennett Denise Richardson | University of Cincinnati Medical Centre |
| Giles Peek | University of Florida |
| Lovkesh Arora Kristina Rappapport Kristina Rudolph Zita Sibenaller Lori Stout Alicia Walter | University of Iowa |
| Daniel Herr Nazli Vedadi | University of Maryland - Baltimore |
| Robert Bartlett | University of Michigan Medical Center |
| Antonio Pesenti | University of Milan |
| Shaun Thompson Julie Hoffman Xiaonan Ying Bailey Williams Emely Sanchez Chika Akwani | University of Nebraska Medical Centre |
| Ryan Kennedy | University of Oklahoma Health Sciences Centre (OU) |
| Muhammed Elhadi | Faculty of Medicine, University of Tripoli |
| Matthew Griffee  Mary Mone Anna Ciullo Yuri Kida | University of Utah Hospital |
| Ricard Ferrer Roca JordI Riera Sofia Contreras Cynthia Alegre | Vall d'Hebron University Hospital, Barcelona |
| Christy Kay Irene Fischer Elizabeth Renner | Washington University in St. Louis/ Barnes Jewish Hospital |
| Hayato Taniguci | Yokohama City University Medical Center |
| James Lee Daniel Plotkin Barbara Wanjiru Citarella Laura Merson | ISARIC, Centre for Tropical Medicine and Global Health, University of Oxford, Oxford, UK |

**Collaborators**

| **Prefix/First Name/Last Name** | **Site Name** |
| --- | --- |
| Emma Hartley | Aberdeen Royal Infirmary (Foresterhill Health Campus) |
| Bastian Lubis | Adam Malik Hospital |
| Takanari Ikeyama | Aichi Childrens Health and Medical Center |
| Ameen Alhamad | Aleppo University Hospital |
| Mohamed Fathi | Al-Hawari Center of Surgical Speciality |
| Mohammed Maher Hadhoud | Al Menshawy General Hospital |
| Hasan Alhouri | Al-Mouwasat University Hospital - Damascus |
| Ahmed Mechi | Al-Sader Teaching Hospital, Al-Najaf |
| Mohammed Saleh Alyasiri | Alshifa Center Medical City |
| Muhammed Zainab Alghali Elsaid | Alshuhada Hospital |
| Dr Hamza Shahla | Al-Thawra Teaching Hospital in Albayda |
| Balu Bhaskar | American Hospital |
| Jae-Seung Jung | Anam Korea University Hospital |
| Shay McGuinness | Auckland City Hospital |
| Glenn Eastwood | Austin Hospital |
| Sandra Rossi Marta  Fabio Guarracino | Azienda Ospedaliero Universitaria Parma |
| Stacy Gerle | Banner University Medical Centre |
| Emily Coxon | Baptist Health Louisville |
| Bruno Claro | Barts Hospital |
| Mahmoud Eleisawy | Benha University Hospital |
| Hasnaa Osama | Beni-Suef University Hospital |
| Daniel Loverde | Billings Clinic |
| Namrata Patil | Brigham and Women’s Hospital |
| Vieri Parrini | Borgo San Lorenzo Hospital |
| Angela McBride | Brighton and Sussex Medical School |
| Kathryn Negaard | Brooke Army Medical Centre |
| Angela Ratsch | Bundaberg Hospital |
| Ahmad Abdelaziz | Cairo University Hospital |
| Juan David Uribe | Cardio VID |
| Adriano Peris | Careggi Hospital |
| Mark Sanders | Cedar Park Regional Medical Center |
| Dominic Emerson | Cedars-Sinai Medical Centre |
| Muhammad Kamal | Cengkareng Hospital |
| Hamza Faida | Centre Hospitalier Universitaire Ibn Sina Rabat |
| Pedro Povoa | Centro Hospitalar de Lisboa |
| Roland Francis | Charite-Univerrsitatsmedizi n Berlin |
| Ali Cherif | Charles Nicolle University Hospital |
| Sunimol Joseph | Children’s Health Ireland (CHI) at Crumlin |
| Matteo Di Nardo | Children’s Hospital Bambino Gesù |
| Micheal Heard | Children's Healthcare of Atlanta – Egleston Hospital |
| Kimberly Kyle | Children's Hospital – Los Angeles |
| Ray A Blackwell | Christiana Care Health System's Centre for Heart and Vascular Health |
| Amel OUYAHIA | CHU - Chu Saadna Abdenour De Sétif - Sétif |
| Michael Piagnerelli  Patrick Biston | CHU de Charleroi |
| Hye Won Jeong | Chungbuk National University Hospital |
| Reanna Smith | Cincinnati Children's |
| Yogi Prawira | Cipto Mangunkusumo Hospital |
| Giorgia Montrucchio | Città della Salute e della Scienza Hospital – Turin, Italy |
| Arturo Huerta Garcia | Clínica Sagrada Família |
| Nahikari Salterain | Clinica Universidad de Navarra |
| Bart Meyns | Collaborative Centre Department Cardiac Surgery, UZ Leuven |
| Muhammed Elnasser | Damascus Hospital |
| Marsha Moreno | Dignity Health Medical Group- Dominican |
| Rajat Walia | Dignity Health St. Joseph's Hospital and Medical Center (SJHMC) |
| Amit Mehta | Doernbecher Children’s Hospital |
| Annette Schweda | Donaustauf Hospital |
| Melissa Williams | Duke University Hospital (Durham) |
| Emad Amkhatirah | Elmarj Teaching Hospital |
| Kyung Hoon Kim | Eunpyeung St Mary's Hospital |
| Alexandra Assad | Fluminense Federal University |
| Estefania Giraldo | Fundación Clinica Shaio (Shaio Clinic) |
| Wojtek Karolak | Gdansk Medical University |
| Martin Balik | General University Hospital |
| Elizabeth Pocock | George Washington University Hospital |
| Akram Mohamed | Gharyan Central Hospital |
| Evan Gajkowski | Giesinger Medical Centre |
| Kanamoto Masafumi | Gunma University Graduate School of Medicine |
| Nicholas Barrett | Guy's and St Thomas NHS Foundation Trust Hospital |
| Yoshihiro Takeyama | Hakodate City Hospital |
| Sunghoon Park | Hallym University Sacred Heart Hospital |
| Faizan Amin | Hamilton General Hospital |
| Fina Meilyana Andriyani | Hasan Sadikin Hospital (Paediatric) |
| Serhii Sudakevych | Heart Institute Ministry of Health of Ukraine |
| Janos Schnur | Heim Pál National Pediatric Institute |
| Angela Ratsch | Hervey Bay Hospital |
| Magdalena Vera | Hospital Clinico de la Pontificia Universidad Catolica |
| Rodrigo Cornejo | Hospital Clinico de la Universidad de Chile |
| Patrícia Schwarz  Ana Carolina Mardini | Hospital de Clínicas de Porto Alegre |
| Thais de Paula | Hospital Felicio Rocho |
| Ary Serpa Neto | Hospital Israelita Albert Einstein |
| Andrea Villoldo | Hospital Privado de Comunidad |
| Alexandre Siciliano Colafranceschi | Hospital Pro Cardíaco |
| Alejandro Ubeda Iglesias | Hospital Punta de Europa |
| Juan Granjean | Hospital Regional de Valdivia |
| Lívia Maria Garcia Melro  Giovana Fioravante Romualdo | Hospital Samaritano Paulista |
| Diego Gaia | Hospital Santa Catarina |
| Helmgton Souza | Hospital Santa Marta |
| Filomena Galas | Hospital Sirio Libanes |
| Rafael Máñez Mendiluce | Hospital Universitario de Bellvitge |
| Alejandra Sosa | Hospital Universitario Esperanza (Universidad Francisco Marroquin) |
| Ignacio Martinez | Hospital Universitario Lucus Augusti |
| Hiroshi Kurosawa | Hyogo Prefectural Kobe Children's Hospital |
| Mohammad Badr Almoshantaf | Ibn Al-Nafees Hospital |
| Juan Salgado | Indiana University Health |
| Beate Hugi-Mayr | Inselspital University Hospital |
| Eric Charbonneau | Institut Universitaire de Cardiologie et de Pneumologie de Quebec - Universite Laval |
| Vitor Salvatore Barzilai | Instituto de Cardiologia do Distrito Federal - ICDF |
| Veronica Monteiro | Instituto de Medicina Integral . Fernando Figueira (IMIP) |
| Rodrigo Ribeiro de Souza | Instituto Goiano de Diagnostico Cardiovascular (IGDC) |
| Michael Harper | INTEGRIS Baptist Medical Center |
| Nidhal Siddig | Jabra Hospital, Khartoum |
| Hiroyuki Suzuki | Japan Red Cross Maebashi Hospital |
| Celina Adams | John C Lincoln Medical Centre |
| Jorge Brieva | John Hunter Hospital |
| Almu'atasim Khamees | Jordan University Hospital |
| Fadi Graige | Kalamoon Hospital |
| Moh Supriatna | Kariadi Hospital Semarang |
| George Nyale | Kenyatta National Hospital (KNH) |
| Faisal Saleem Eltatar | King Abdullah Medical City |
| Jihan Fatani | King Abdullah Medical City Specialist Hospital |
| Husam Baeissa | King Abdullah Medical Complex |
| Ayman AL Masri | King Salman Hospital NWAF |
| Ahmed Rabie | King Saud Medical City |
| Mok Yee Hui | KK Women's and Children's Hospital |
| Masahiro Yamane | KKR Medical Center |
| Hanna Jung | Kyung Pook National University Hospital |
| Ayorinde Mojisola Margaret | Lagos University Teaching Hospital |
| Newell Nacpil | Lung Center of the Philippines |
| Katja Ruck | Luxembourg Heart Center |
| Rhonda Bakken | M Health Fairview |
| Claire Jara | Maine Medical Centre (Portland Maine) |
| Tim Felton | Manchester University NHS Foundation Trust - Wythenshawe |
| Samar Tharwat | Mansoura University Hospital |
| Lorenzo Berra | Massachusetts General Hospital |
| Bobby Shah | Medanta Hospital |
| Arpan Chakraborty | Medica Super speciality Hospital |
| Monika Cardona | Medical University of South Carolina |
| Gerry Capatos | Mediclinic Parkview Hospital Dubai |
| Bindu Akkanti | Memorial Hermann - Texas Medical Centre |
| Abiodun Orija | Memorial Regional Hospital (Hollywood Florida) |
| Harsh Jain | Mercy Hospital of Buffalo |
| Asami Ito | Mie University Hospital |
| Mohamed Muftah | Misurata Medical Center |
| Brahim Housni | Mohammed VI University Hospital |
| Amer Aldhalia | Nasar City Hospital for Insurance |
| Sennen Low | National Centre for Infectious Diseases |
| Koji Iihara | National Cerebral and Cardiovascular Center |
| Joselito Chavez | National Kidney and Transplant Institute |
| Kollengode Ramanathan | National University Hospital, Singapore |
| Gustavo Zabert | National University of Comahue |
| Krubin Naidoo | Nelson Mandela Children's Hospital |
| Ian Seppelt | Nepean Hospital |
| Marlice VanDyk  Sarah MacDonald | Netcare Unitas ECMO Centre |
| Shingo Ichiba | Nippon Medical School Hospital |
| Wael Hafez | NMC Royal Hospital, Abu Dhabi |
| Randy McGregor | Northwestern Medicine |
| Teka Siebenaler | Norton Children's Hospital |
| Hannah Flynn | Novant Health (NH) Presbyterian Medical Centre |
| Kristi Lofton | Ochsner LSA Health Shreveport |
| Toshiyuki Aokage | Okayama University Hospital |
| Bakar Kvirkvelia | Open Heart 5^th^ Clinical Hospital, Tbilisi |
| Kazuaki Shigemitsu | Osaka City General Hospital |
| Andrea Moscatelli | Ospedale Gaslini |
| Giuseppe Fiorentino | Ospedali dei Colli |
| Matthias Baumgaertel | Paracelsus Medical University Nuremberg |
| Serge Eddy Mba | Parirenyatwa General Hospital |
| Jana Assy | Pediatric and Neonatal Cardiac Intensive Care at the American University |
| Amelya Hutahaean | Pelni Hospital |
| Holly Roush | Penn State Heath S. Hershey Medical Centre |
| Kay A Sichting | Peyton Manning Children's Hospital |
| Francesco Alessandri | Policlinico Umberto, Sapienza University of Rome |
| Debra Burns | Presbyterian Hospital, New York/ Weill Cornell Medical Centre |
| Taha Husayn Alkhubouli | Preventive Medicine Hospital |
| Ahmad Nasrallah | Prince Hamza Hospital- Amman |
| Ahmed Rabie | Prince Mohammed bin Abdulaziz Hospital |
| Gavin Salt | Prince of Wales |
| Carl P. Garabedian | Providence Sacred Heart Children's Hospital |
| Jonathan Millar  Malcolm Sim | Queen Elizabeth II University Hospital |
| Adrian Mattke | Queensland Children’s Hospital |
| Danny McAuley | Queens University of Belfast |
| Jawad Tadili | Rabat University Hospital |
| Tim Frenzel | Radboud University Medical Centre |
| Yaron Bar-Lavie | Rambam Hospital |
| Aaron Blandino Ortiz | Ramón y Cajal University Hospital |
| Jackie Stone | Rapha Medical Centre |
| Alexis Tabah | Redcliffe Hospital |
| Antony Attokaran | Rockhampton Hospital |
| Michael Farquharson | Royal Adelaide Hospital |
| Brij Patel | Royal Brompton & Harefield NHS Foundation Trust |
| Derek Gunning | Royal Columbian Hospital |
| Kenneth Baillie | Royal Infirmary Edinburgh |
| Safia Adem | Sabha Medical Center |
| Pia Watson | Sahlgrenska University Hospital |
| Kenji Tamai | Saiseikai Yokohamashi Tobu Hospital |
| Gede Ketut Sajinadiyasa  Dyah Kanyawati | Sanglah General Hospital |
| Marcello Salgado | Santa Casa de Misericordia de Juiz de Fora |
| Assad Sassine | Santa Casa de Misericórdia de Vitoria |
| Bhirowo Yudo | Sardjito Hospital |
| Scott McCaul | Scripps Memorial Hospital La Jolla |
| Bongjin Lee | Seoul National University Children's Hospital |
| Sang Min Lee | Seoul National University Hospital |
| Arnon Afek | Sheba Medical Center |
| Yoshiaki Iwashita | Shimane University Hospital |
| Hammad Fadlalmola | Soba University Hospital |
| Bambang Pujo Semedi  Neurinda Permata Kusumastuti | Soetomo General Hospital (FK UNAIR) |
| Noureldin Mohamed Mansour | Souad Kafafi University Hospital |
| Jack Metiva | Spectrum Health Western Governors University |
| Nicole Van Belle | St. Antonius Hospital |
| Ignacio Martin-Loeches | St James’s University Hospital |
| Dr Mohammed Al-Sadawi | Stony Brook University |
| Cenk Kirakli | Suat Seren Chest Diseases and Surgery Practice and Training Centre |
| Al-Touny Shimaa | Suez Canal University Hospitals |
| Abusalama Abdurraouf | Swani Health Isolation Center |
| Lenny Ivatt | Swansea Hospital |
| Saad Moharam | Tanta University Hospital |
| Chia Yew Woon | Tan Tock Seng Hospital |
| Hyun Mi Kang | The Catholic University of Seoul St Mary Hospital |
| Timothy Smith | The Christ Hospital |
| Erskine James | The Medical Centre Navicent Health |
| Nawar Al-Rawas | Thomas Jefferson University Hospital |
| Abdulrahman Almjersah | Tishreen University Hospital |
| Yudai Iwasaki | Tohoku University |
| Hamza Ashour | Traghen Hospital |
| Hussein Embarek | Tripoli University Hospital |
| Kenny Chan King-Chung | Tuen Mun Hospital |
| Vadim Gudzenko | UCLA Medical Centre (Ronald Regan) |
| Beate Hugi-Mayr | Universitätsspital Bern, Universitätsklinik für Herz- und Gefässchirurgie |
| Fabio Taccone | Universite Libre de Bruxelles |
| Fajar Perdhana | University Airlangga Hospital (Adult) |
| Yoan Lamarche | University de Montreal (Montreal Heart Institute) |
| Joao Miguel Ribeiro | University Hospital CHLN |
| Nikola Bradic | University Hospital Dubrava |
| Klaartje Van den Bossche | University Hospital Leuven |
| Oude Lansink | University Medical Center Groningen |
| Gurmeet Singh | University of Aberta (Mazankowski Heart Institute) |
| Gerdy Debeuckelaere | University of Antwerp |
| Henry T. Stelfox | University of Calgary and Alberta Health Services |
| Cassia Yi | University of California at San Diego |
| Jennifer Elia | University of California, Irvine |
| Thomas Tribble | University of Kentucky Medical Center |
| Shyam Shankar | University of Missouri |
| Raj Padmanabhan | University of Pittsburgh Medical Centre |
| Bill Hallinan | University of Rochester Medical Centre (UR Medicine) |
| Luca Paoletti | University of South Carolina |
| Yolanda Leyva | University of Texas Medical Branch |
| Tatuma Fykuda | University of the Ryukyus |
| Jenelle Badulak | University of Washington in Seattle |
| Jillian Koch | University of Wisconsin & American Family Children's Hospital |
| Lisa Janowaik | UTHealth (University of Texas) |
| Amy Hackman | UT Southwestern |
| Deb Hernandez | Valley Children's Hospital (Madera) |
| Jennifer Osofsky | Vassar Brothers Medical Center (VBMC) |
| Katia Donadello | Verona Integrated University Hospital |
| Aizah Lawang | Wahidin Sudirohusodo Hospital |
| Josh Fine | WellSpan Health - York Hospital |
| Benjamin Davidson | Westmead Hospital |
| Andres Oswaldo Razo Vazquez | Yale New Haven Hospital |
| Ibrahim Abdehaleem | Zagazig University Hospital |
